# The Effect of β-Carotene, Tocopherols and Ascorbic Acid as Anti-Oxidant Molecules on Human and Animal In Vitro/In Vivo Studies: A Review of Research Design and Analytical Techniques Used

**DOI:** 10.3390/biom12081087

**Published:** 2022-08-07

**Authors:** Krystian Miazek, Karolina Beton, Agnieszka Śliwińska, Beata Brożek-Płuska

**Affiliations:** 1 Laboratory of Laser Molecular Spectroscopy, Institute of Applied Radiation Chemistry, Lodz University of Technology, Wroblewskiego 15, 93-590 Lodz, Poland; 2 Department of Nucleic Acid Biochemistry, Medical University of Lodz, 251 Pomorska Str., 92-213 Lodz, Poland

**Keywords:** oxidative stress, reactive oxygen species, antioxidants, biomarkers, raman spectroscopy and imaging

## Abstract

Prolonged elevated oxidative stress (OS) possesses negative effect on cell structure and functioning, and is associated with the development of numerous disorders. Naturally occurred anti-oxidant compounds reduce the oxidative stress in living organisms. In this review, antioxidant properties of β-carotene, tocopherols and ascorbic acid are presented based on in vitro, in vivo and populational studies. Firstly, environmental factors contributing to the OS occurrence and intracellular sources of Reactive Oxygen Species (ROS) generation, as well as ROS-mediated cellular structure degradation, are introduced. Secondly, enzymatic and non-enzymatic mechanism of anti-oxidant defence against OS development, is presented. Furthermore, ROS-preventing mechanisms and effectiveness of β-carotene, tocopherols and ascorbic acid as anti-oxidants are summarized, based on studies where different ROS-generating (oxidizing) agents are used. Oxidative stress biomarkers, as indicators on OS level and prevention by anti-oxidant supplementation, are presented with a focus on the methods (spectrophotometric, fluorometric, chromatographic, immuno-enzymatic) of their detection. Finally, the application of Raman spectroscopy and imaging as a tool for monitoring the effect of anti-oxidant (β-carotene, ascorbic acid) on cell structure and metabolism, is proposed. Literature data gathered suggest that β-carotene, tocopherols and ascorbic acid possess potential to mitigate oxidative stress in various biological systems. Moreover, Raman spectroscopy and imaging can be a valuable technique to study the effect of oxidative stress and anti-oxidant molecules in cell studies.

## 1. Introduction

Oxidative stress (OS) is defined by the production and accumulation of Reactive Oxygen Species (ROS) in biological systems above the capacity of cells and tissues to neutralize ROS presence to a safe level [1]. Reactive Oxygen Species (ROS) are molecular oxygen (O_2_)-derived characterized by high reactivity towards other molecules. ROS comprise free radicals such as superoxide anion radical (O_2_^•−^) and hydroxyl radical (•OH), and nonradical molecules such as singlet oxygen (^1^O_2_) and hydrogen peroxide (H_2_O_2_). Singlet oxygen (^1^O_2_) is the exited state of ground state triplet molecular oxygen (^3^O_2_), and is generated via absorption of energy sufficient to reverse the spin of one of its unpaired electrons what leads to the formation of singlet state where two electrons possess opposite spin [2]. Superoxide anion radical (O_2_^•^^−^) is generated by one-electron reduction of molecular oxygen (O_2_), what results in the production of a charged ionic species (O_2_^•−^) possessing a single unpaired electron and a negative charge [3]. Hydrogen peroxide (H_2_O_2_), a compound possessing an oxygen-oxygen single bond [4], is generated from dismutation of O_2_^•−^ [3]. H_2_O_2_ can be decomposed via Fenton reaction to OH^−^ and •OH with Fe^2+^ oxidation, or can react with O_2_^•−^ to form •OH, OH^−^ and O_2_ [2]. Hydroxyl radical (•OH) possesses a single unpaired electron and is generated by oxidation of water (H_2_O) or hydroxide ions (OH^−^), or by decomposition of hydrogen peroxide (H_2_O_2_) via Fenton reaction [5,6].

**Figure 1:**
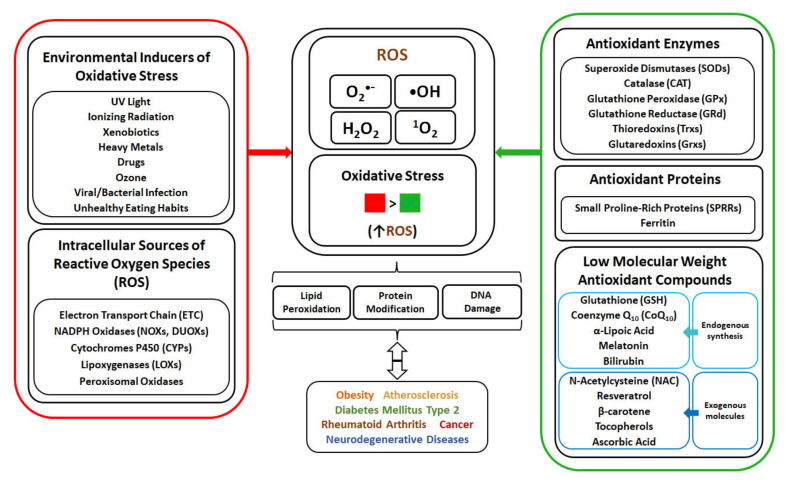
The overview of oxidative stress (OS) generation in human, with various environmental factors as the cause of OS, with different ROS types and different intracellular sites for ROS generation, OS relation with the disease’s occurrence, and anti-oxidant mechanisms involving enzymatic and non-enzymatic molecules.

In living organisms, ROS are produced during indigenous metabolism to serve as signalling molecules, but can be also generated at high concentrations due to exposure to environmental stress factors (radiation, pollution, pathogens, etc.) [7]. Generated ROS include singlet oxygen (^1^O_2_), superoxide anion radical (O_2_^•^^−^), hydrogen peroxide (H_2_O_2_) and hydroxyl radical (•OH), and are characterized by different reactivity. ^1^O_2_ is a highly reactive form of oxygen possessing two electrons with opposite spins, and oxidizing molecules via reaction with double bonds [8,9]. Although O_2_^•^^−^ is not a strong oxidant, it can undergo dismutation to H_2_O_2_ that can decompose to form a •OH, one of the strongest oxidants. O_2_^•−^ can also react with nitric oxide radical to form a peroxynitrite, also considered a powerful oxidant [8]. The elevated production of ROS and oxidative stress cause detrimental effect on organisms due to the damage of cellular macromolecules, with oxidation of lipids, proteins and nucleic acids. In human, the occurrence and development of many diseases such as obesity, atherosclerosis, diabetes mellitus type 2 (DMT2), rheumatoid arthritis, cancer and neurodegenerative diseases are associated with oxidative stress [10]. To cope with oxidative stress, organisms developed defence mechanisms (Figure 1) involving anti-oxidant enzymes, as well as non-enzymatic molecules. The human enzymatic anti-oxidant mechanisms include enzymes such as superoxide dismutase (SOD), catalase (CAT), glutathione peroxidase (GPx), glutathione reductase (GRd) and thioredoxins (Trx), with glutathione (GSH) serving as electron donor and used in reactions catalysed by GPx. The non-enzymatic anti-oxidant system in human body is composed of anti-oxidant proteins, such as proline-rich proteins and ferritin, and low-weight anti-oxidant molecules synthesized indigenously, such as coenzyme Q_10_, α-lipoic acid, melatonin and bilirubin. Moreover, addition of external low-weight anti-oxidant molecules, such as N-acetylcysteine, resveratrol, β-carotene, tocopherols and ascorbic acid, can strengthen the capability of indigenous defence system against OS.

The mitigating effect of β-carotene, tocopherols and ascorbic acid against oxidative stress is presented in this article. β-carotene classified as provitamin A, ascorbic acid named as vitamin C and tocopherols possessing activity of vitamin E, are exogenous anti-oxidant molecules with ROS-scavenging properties that are considered useful in preventing oxidative stress in mammalian cells and organisms. In this review, anti-oxidant properties of β-carotene, tocopherols and ascorbic acid to alleviate oxidative stress based on in vitro, in vivo as well as populational studies, are described. Moreover, different analytical techniques (spectrophotometric, fluorometric, chromatographic, immune-enzymatic, spectroscopic) used commonly to assess the OS presence and anti-oxidant properties of tested molecules, are compared and discussed.

Overall, this review provides a broad description regarding the effect of β-carotene, ascorbic acid and tocopherols as potential antioxidants to mitigate oxidative stress in human and animal studies, in terms of research designs, experimental outcomes and analytical techniques applied.

## 2. Environmental Factors as Inducers of Oxidative Stress

Environmental factors, such as radiation (ultraviolet, ionizing), tobacco smoke, xenobiotics and other pollutants as well as infections, are inducers of ROS generation and oxidative stress (Figure 1).

Ultraviolet (UV) light, a part of electromagnetic radiation spectrum with wavelength range of 100 nm to 400 nm, is divided into UVA (320–400 nm), UVB (290–320 nm) and UVC (220–290 nm) light. The sources of UV light can be natural (sun) or artificial (phototherapy, sunbeds, arc welding, germicidal lamps). UV radiation can be absorbed by cellular components resulting in their conversion into the exited state, performing chemical reactions with ROS generation. UV rays can be also absorbed by photosensitizers (endogenous and exogenous), which in their exited state, can abstract hydrogen to form free radicals, or can transfer energy with O_2_ to form ^1^O_2_ [11]. UV-induced reactive oxygen species contribute to the damaging effect of UV towards the skin [12].

Ionizing radiation (IR), including gamma and X rays, is the radiation capable of inducing atom ionization via reactions of electron ejection [13]. The sources of IR are radiotherapy, nuclear accidents, atomic bombing, soil radioisotopes and cosmic rays [14,15]. Ionizing radiation causes radiolysis of water and linear energy transfer (LET)-mediated generation of reactive chemical species (•OH, H_2_O_2_, O_2_^•−^) that can damage macromolecules (nucleic acids, proteins, lipids) [16]. IR-generated reactive species cause cellular damage with detrimental effect on living organisms [13].

Xenobiotics, represented by polycyclic aromatic hydrocarbons and pesticides, are organic substances being foreign to living organisms and possessing structural abilities to induce oxidative stress in organs and tissues. Polycyclic aromatic hydrocarbons (PAHs) are formed during incomplete combustion processes of fuels and from traffic emissions [17]. PAHs undergo oxidation into phenolic intermediates which are converted via semiquinone anion radicals into quinones, with generation of superoxide anion radicals and H_2_O_2_ [18,19]. Pesticides (herbicides, insecticides, fungicides, etc.), used in agriculture for crop protection, can be found as contaminants in air, water and food. The mode of oxidative action of pesticides, with herbicide paraquat as an example, is the induction of mitochondrial damage and the redox cycle involving quaternary ammonium nitrogen atoms and a bipirydyl ring in paraquat structure, what leads to production of ROS and paraquat radicals [20].

Heavy metals (HMs), such as Pb, Cd, Cr, Hg and As, are contaminants released from industry (effluents, waste product storage) to the surroundings (atmosphere, soil, water) and affecting human beings [21]. Accumulation of HMs ions in body induces oxidative stress within a range of mechanisms, including inhibition of antioxidant enzyme expression (by Cd), interaction with cofactors and/or disulphide bonds in antioxidant enzymes (e.g., Pb, Hg), haemoglobin autooxidation (by Pb^2+^), binding to sulfhydryl groups (−SH) and reducing thiol pools (through Cd^2+^ or Hg^2+^), generating glutathione-thiyl radicals (via Cr(VI)), changing the oxidation state of HMs with formation of H_2_O_2_ and hydroxyl radicals (through Cr or As), affecting calcium homeostasis and stimulating oxidative enzymes (by Hg), cytokine-mediated ROS generation (via Cd^2+^), and others [18,22,23]. Heavy metal-induced oxidative stress and macromolecules modification/degradation are the cause of many diseases, amongst which are cancer, cardiovascular disease, neurological disorders and chronic inflammation [24]. 

Drugs used for illness treatment are also the source of ROS generation in human body [25]. Anti-neoplastic agents, such as doxorubicin and cisplatin, are used for the treatment of different types of cancer. Doxorubicin, a representative of anthracycline antibiotics, generates ROS by undergoing mitochondrial reductase-mediated one-electron reduction to anthracycline semiquinone free radicals, that can react with O_2_ to form O_2_^•−^ or H_2_O_2_. Doxorubicin can also interact with Fe^3+^ to form Fe^2+^-doxorubicin free radical, that can reduce oxygen. Oxidative stress induction is the mechanism of doxorubicin cardiotoxicity [25]. Cisplatin, a platinum containing drug, was reported to increase the ROS level, via NAPDH oxidase or xanthine oxidase [25,26]. Oxidative stress induction in the proposed mechanism of cisplatin nephrotoxicity and ototoxicity [26,27].

Smoking, with cigarette smoke containing nicotine, ammonia, acrolein, phenols, acetaldehyde, polycyclic aromatic hydrocarbons, polyphenols, hydrogen cyanide, heavy metals, etc., is another source of ROS production and oxidative stress occurrence [28,29]. The tobacco smoke contains gas phase and tar phase. The gas phase contains short-lived radicals, superoxide anion and nitric oxide, which react together to form highly reactive peroxynitrite. The tar phase contains stable semiquinone radicals and iron (Fe^2+^). Semiquinone radicals reduce O_2_ to O_2_^•−^, that can dismutate into H_2_O_2_, which in turn can react with Fe^2+^ via Fenton reaction to form •OH [29,30]. Oxidative stress is considered as a crucial factor in the pathogenesis of smoking-related disorders, such as lung cancer, chronic obstructive pulmonary disease and atherosclerosis [31]. 

Ozone (O_3_) is a gaseous tropospheric pollutant, generated through reactions between intense solar radiation and pollutants (nitric oxides, sulphur oxides, carbon oxides, volatile organic compounds) produced from combustion of fossil fuels [32,33]. Contact of O_3_ with biological matrix results in creation of H_2_O_2_ and lipids oxidation products [34]. Ozone exposure-induced oxidative stress is associated with neurodegenerative diseases [33,35].

The infections by viruses or bacteria can be also the cause of oxidative stress in human body. The body can be infected by viruses, such as DNA and RNA viruses, which enter and replicate inside host cells [36,37]. ROS are generated during viral infection via inducing activation of phagocytes [36] or via mediation of viral proteins expressed in host cells to support viral life cycle [37]. Oxidative stress occurring during bacterial infection is described for *Helicobacter pylori*, a gram negative, stomach-infecting bacterium. *H. pylori* infection results in oxidative stress via the immune and gastric epithelial cells producing ROS in an attempt to kill the bacteria, and via bacterial virulence factors inducing epithelium cellular responses and ROS generation [38].

Unhealthy dietary patterns, based on overconsumption of high-carbohydrate and high-fat food, are associated with increased risk of overweight and obesity occurrence and development of diabetes mellitus type 2 (DMT2) and cardiovascular diseases. High-fat or high-carbohydrate diets results in the elevated influx of substrates into mitochondrial respiration and increased donation of electrons to electron transport chain, leading to the electron leakage at complex III and elevated superoxide (O_2_^•−^) generation. [39]. NADPH oxidase (NOX), an enzyme converting molecular oxygen to its superoxide radical, is also involved in nutrient-based ROS generation [40]. High-calorie diets may alter oxygen metabolism and are considered as one of the main factors leading to excessive ROS production [41].

## 3. Metabolic Pathways as Sources of ROS Generation in Cells

The main endogenous sources of ROS are enzymes of mitochondrial respiratory chain and nicotinamide adenine dinucleotide phosphate (NADPH) oxidase enzymes, whereas other sources of ROS are cytochromes P450, lipoxygenases and peroxisomal enzymes (Figure 1).

Electron transport chain (ETC), embedded within mitochondrial inner membranes, are sources of ROS production. The ETC includes transmembrane protein complexes NADH: ubiquinone oxidoreductase (Complex I), succinate dehydrogenase (Complex II), ubiquinol: cytochrome c oxidoreductase (Complex III) and cytochrome c oxidase (Complex IV), as well as mobile electron transporters (ubiquinone, cytochrome c). ETC participates in the process of oxidative phosphorylation (OXPHOS), where O_2_ is converted to H_2_O and adenosine triphosphate (ATP) is produced. In brief, electrons from mitochondrial matrix tricarboxylic acid (TCA) cycle are donated via NADH to Complex I and via FADH_2_ to Complex II, and are transferred from Complex I and II to ubiquinone (Q) that undergoes reduction to ubiquinol (QH_2_). Ubiquinol, carrying electrons, becomes re-oxidized via Q-cycle in Complex III, that passes electrons via cytochrome c (CytC) to Complex IV. Subsequently, complex IV transfers electrons to O_2_ as electron acceptor with generation of H_2_O. The electron flow (CI&CII→QH_2_→CIII→CytC→CIV→H_2_O) is coupled with the pumping of protons (H^+^) from mitochondrial matrix into the intermembrane space by complexes I, III and IV. The proton gradient formed across the mitochondrial inner membrane is used by complex V (ATP synthase) to produce (ATP). ETC can produce reactive oxygen species due to the leakage of electrons from Complex I, II and III, resulting in one-electron reduction of oxygen to O_2_^•^^−^. O_2_^•−^, formed via Complex I and II, occur in the matrix, whereas Complex III O_2_^•−^ is released into both the matrix and intermembrane space. O_2_^•−^ is dismutated to H_2_O_2_ by SOD2 in the matrix and by SOD1 in the intermembrane space [7,42,43]. Complex IV is not directly involved in ROS production, but its activity can affect the overall electron flow, with an impact on the electron leakage by previous complexes [44]. It is estimated that 0.2–2% of the electrons passing through the ETC leak out and interact with oxygen to produce ROS [43].

The NADPH oxidases (NOX) are multi-subunit enzymes localized in membranes. NOXs transfer electrons across biological membranes to oxygen, thereby generating superoxide anion radical and or hydrogen peroxide. The mechanism of catalysis involves transfer of two electrons from NADPH through FAD domain and two heme prosthetic groups in enzyme structure to O_2_ [45]. The NOX family comprises of seven isoforms (NOX1, NOX2, NOX3, NOX4, NOX5, DUOX1 and DUOX2) [46,47] expressed in different tissues throughout the body. NOX1 is highly expressed in the epithelial cells of the gastrointestinal tract [48], and is also present in prostate, uterus, vascular cells [49] and erythrocytes [50]. NOX2 is typically expressed in phagocytic cells, but also in endothelial cells, cardiomyocytes, hematopoietic stem cells and platelets [51]. NOX3 is expressed in the inner ear [48], and is also found in low abundance in the brain, lung and in fetal tissue [49]. NOX4 is commonly distributed in human tissues, and highly expressed in kidney, osteoclasts, fibroblasts and endothelial cells [47,52]. NOX5 is expressed in testis and lymphoid tissue, but also in placenta, uterus, stomach, skeletal muscle, hepatocytes, cells of the cardiovascular system (cardiomyocytes, endothelial and vascular smooth muscle cells) [49] and erythrocytes [50]. DUOX1 and DUOX2 are highly expressed in thyroid gland [49]. O_2_^•−^ is generated by NOX1-3 and NOX5, while H_2_O_2_ is produced by DUOX1-2 and NOX4 [53]. 

Cytochromes P450 (CYPs) are enzymes localized primarily in the endoplasmic reticulum and found mostly in liver and intestinal tissues [54]. CYPs are enzymes containing a heme prosthetic group in the form of iron protoporphyrin IX and participating in the metabolism of xenobiotics and endogenous compounds. CYPs possess monooxygenase activity and catalyse the incorporation of one atom of oxygen from O_2_ to organic substrate and reduction of the second oxygen atom to water. The catalytic mechanism of CYPs relies on redox reaction cycle of cysteine-bound iron atom involving substrate binding to ferric iron and reduction of Fe^3+^ to Fe^2+^, followed by O_2_ binding to iron and formation of complex (Fe^2+^-O_2_) that undergoes one-electron reduction (Fe^2+^-O_2_^−^) and protonation to cleave O-O bond and release H_2_O, and the transfer of oxygen atom from FeO^3+^ complex to substrate, with the formation of mono-oxygenated substrate and regeneration of Fe^3+^ in CYP. During CYP monooxygenase cycle, reactive oxygen species can be produced either via releasing of superoxide radical with its dismutation to H_2_O_2_ or direct releasing H_2_O_2_ [55,56]. 

Lipoxygenases (LOXs) are nonheme, iron-containing enzymes that catalyse oxygenation of arachidonic acid (AA) to hydroperoxyeicosatetraenoic acids (HPETEs), which can be further converted to hydroxyeicosatetraenoic acids (HETEs), leukotrienes, lipoxins and hepoxilins [57,58]. In human, there are 5-LOX, 12-LOX, and 15-LOX, that catalyse the insertion of oxygen molecule at C-5, C-12 and C-15 of AA, respectively [59,60]. LOX-generated AA metabolites can induce ROS generation in various cells via NOX upregulation [59].

Peroxisomes are single-membrane subcellular organelles, found in all eukaryotic cells, participating in the range of metabolic pathways such as α- and β-oxidation of very long fatty acids, the synthesis of bile acids, detoxification of glyoxylate and H_2_O_2_ metabolism [61,62]. Peroxisomal enzymes, such as xanthine oxidase and acyl-CoA oxidases, are involved in ROS generation [63]. Xanthine oxidase (XO) participates in the catabolism of purine nucleic acids [64]. XO catalyses the oxidation of hypoxanthine and xanthine with generation of superoxide anion or hydrogen peroxide, respectively, via the monovalent and divalent electron transfer to O_2_ [65]. Acyl-CoA oxidases are enzymes involved in the first step of β-oxidation of different substances, such as CoA-esters of very long-chain fatty acids, branched-chain fatty acids and C27-bile acid intermediates [66]. In human peroxisomes, straight-chain acyl-CoA oxidase, branched-chain acyl-CoA oxidase and pristanoyl-CoA oxidase, are available. Acyl-CoA oxidases catalyse dehydrogenation of acyl-CoA esters, with the Flavin Adenine Dinucleotide (FAD)-mediated transfer of the protons from the β-carbon bond of an acyl-CoA, resulting in generation of *trans*-2-enoyl-CoA esters and FADH_2_. Subsequently, FADH_2_ is regenerated to FAD and hydrogen atoms are transferred to O_2_ to form H_2_O_2_ [67,68].

## 4. Effect of ROS on Cellular Macromolecules

Reactive oxygen species can react with lipids, proteins and nucleic acids (Table 1), the primal macromolecules in cellular structures. ROS cause peroxidation of polyunsaturated fatty acids (PUFAs) with the formation of lipid hydroperoxides, that undergo the cleavage to dialdehydes, such as malondialdehyde (MDA) and glyoxal, and a range of unsaturated aldehydes including 4-hydroxynonenal (4-HNE), crotonaldehyde, 2-propenal (acrolein) and 2-hexenal [69]. ROS can react with amino-acids in protein structure, causing conversion of phenylalanine to *o*- or *m*-tyrosine, tyrosine to di-tyrosine, tryptophan to N-formylkynurenine, leucine to 4- or 5-hydroxyleucine, valine to 3- or 4-hydroxyvaline, as well as oxidation of thiol groups in cysteine and methionine. Aldehydes (e.g., 4-HNE), generated from PUFAs peroxidation, can cause the carbonylation of proteins via addition with S atom of cysteine, N in the imidizole of histidine and N in the amine of lysine [69,70]. ROS, such as hydroxyl radical, can also react with DNA bases (G, T, C, A) and sugar moieties. Hydroxyl radical reacts with C-8 of guanine (G) to generate an 8-hydroxy-7,8-dihydroguanyl radical, that can undergo the oxidation to 8-oxo-7,8-dihydroguanine (8-oxoG), or the reduction to ring-opened 2,6-diamino-4-hydroxy-5-formamidopyrimidine. The products of adenine modifications were reported to be 8-oxo-7,8-dihydroadenine (8-oxoA) and 4,6-diamino-5-formamidopyrimidine. Hydroxyl radical also reacts with C-5 and C-6 in pyrimidines of thymine (T) and cytosine (C), forming 5,6-dihydroxy-5,6-dihydrothymine (T glycol) and 5,6-dihydroxy-5,6-dihydrocytosine (C glycol) [25,71]. Hydroxyl radical can also react with deoxyribose, via abstracting H-atom from C-5 of sugar moiety in DNA, resulting in the creation of C-5-centered radical that forms a bond with C-8 of purine ring in the same nucleotide, thereby yielding 8,5-cyclopurine-2-deoxynucleoside [71]. ROS-mediated oxidation of macromolecules leads to degradation of cellular structures which is associated with metabolic complications and diseases development.

## 5. Oxidative Stress and Diseases Development

Oxidative stress is involved in the occurrence and physiopathology of chronic diseases, such as obesity, atherosclerosis, diabetes mellitus type 2 (DMT2), rheumatoid arthritis, cancer and neurodegenerative diseases [10]. 

Obesity, characterized by an increase in body weight resulting in excessive fat accumulation, represents a public health problem with increasing worldwide prevalence [72,73]. Oxidative stress performs a role in the pathogenesis of obesity by stimulating the deposition of adipose tissue and altering food intake [74]. Oxidative stress is also induced by obesity, as the excess of free fatty acids (FFA) leads to increased FFA oxidation and mitochondrial ROS overproduction. Moreover, the increased release of FA from over-accumulated fat can result in NOX activation and progressed ROS generation [40]. In obesity, the number and size of adipocytes are increased, and secretion of pro-inflammatory molecules is promoted [73]. Oxidative stress occurring in obesity is linked with inflammation and can contribute to the obesity-associated development of diseases, such as atherosclerosis, diabetes and cancer [40].

Atherosclerosis is a chronic inflammatory condition, characterized by gradual accumulation of plaques, composed of fibrous cap, lipid-rich core and calcium, within the artery wall [75]. The rupture of atherosclerotic plaques leads to thrombosis which is the reason of myocardial infarction or stroke [76]. The origin of atherosclerosis is ascribed to hypertension, hypercholesterolaemia, smoking or hyperglycemia that cause the damage of endothelium and the entry of cholesterol-carrying low-density lipoproteins (LDLs) from the blood stream into the epithelium intima [77]. The endothelial cells, smooth muscle cells (SMCs) and macrophages are the source of oxidative stress, that cause the oxidation of LDL particles [78]. Oxidized LDL (Ox-LDL) particles are taken up by macrophages, which are converted into foam cells. The death of foam cells results in the accumulation of cells debris and lipids, and release of proinflammatory cytokines that cause SMCs migration and proliferation. The plaque is progressively formed by agglomerating calcium deposits, SMCs, collagen and foam cells [75,77,79]. 

Diabetes mellitus type 2 (DMT2) is a disease characterized by tissue resistance to insulin, hyperglycemia and decreased secretion of insulin by pancreatic β-cells [80]. In DMT2, the occurrence of OS is ascribed to frequent hyperglycemia, mitochondrial dysfunction and endoplasmic reticulum (ER) stress in β-cells [81], and OS-induced complications of diabetes may include macrovascular (coronary heart diseases, stroke) and microvascular (neuropathy, retinopathy, nephropathy) complications [80].

Rheumatoid arthritis (RA) is an autoimmune disease characterized by joint destruction. Oxidative stress is involved in the pathogenesis of RA, by stimulating inflammation and being stimulated by inflammation, what results in the establishment of synovitis, which causes cartilage and bone damage [82]. 

Cancer is a disease characterized by transformation of normal cells into malignant cells that proliferate in an uncontrolled manner and invade normal tissues and organs, eventually spreading throughout the body. Cancer is a major disease and the second leading cause of mortality worldwide, with more than 277 different types of cancer diseases affecting different parts of body (colon, breast, lung, liver, prostate, brain, skin, bladder, renal, stomach etc.) [83,84,85,86]. Oxidative damage of DNA leads to disruption of genome function, distribution of mutation, selective clonal expansion of the mutated cell and further cancer progression [87,88]. Oxidative stress may be an initiating factor in carcinogenesis and can be also the consequence of cancer development [89].

Neurodegenerative diseases, characterized by progressive dysfunction of neural cells and losses of neurons, include Alzheimer’s disease, Parkinson’s disease and amyotrophic lateral sclerosis [90]. Oxidative stress is one of factors involved in the pathogenesis of neurodegenerative disorders [91]. Neural microenvironment is susceptible to oxidative stress due to high oxygen demand, the abundant presence of redox-active metals, high level of cellular membrane PUFAs and low levels of GSH in the brain [92].

## 6. Anti-Oxidant Mechanisms as a Protection against Oxidative-Stress

Cells possess antioxidant mechanisms, such as enzymes and other proteins, as well as endogenous and exogenous low molecular weight anti-oxidant molecules, to protect cellular structures from oxidative stress and damage (Figure 1). 

### 6.1. Superoxide Dismutase (SOD) and Catalase (CAT)

Superoxide dismutase (SOD) is a metalloenzyme that prevents intracellular O_2_^•^^−^ accumulation by catalysing dismutation of two molecules of O_2_^•−^ to O_2_ and H_2_O_2_. The mechanism of SOD catalytic action is based on the redox cycle of metal ion in the active site, involving metal reduction and oxidation of first O_2_^•−^ to O_2_, followed by metal oxidation and reduction of a second O_2_^•−^ to H_2_O_2_. Three isoforms of SOD: Cu,Zn-SOD (SOD1) homodimer distributed in the cytosol and the mitochondrial intermembrane, Mn-SOD (SOD2) homotetramer located in the mitochondrial matrix and inner membrane, and Cu,Zn-SOD (SOD3) homotetramer anchored to the extracellular matrix, are known to be present in human [93,94]. 

Catalase (CAT) is a tetrameric protein with 4 similar subunits, each containing a ferriprotoporphyrin. CAT catalyses the reduction of 2 molecules of H_2_O_2_ to 2 molecules of H_2_O and one O_2_, within a two-step reaction mode. The first step involves reduction of one H_2_O_2_ molecule with oxidation of heme to an oxyferryl species [porphyrin Fe(IV)-O], having a porphyrin *π*-cation radical. In the second step, a porphyrin radical is reduced by a two-electron transfer from the second H_2_O_2_ molecule to generate the enzyme at resting state [porphyrin Fe(III)] and produce water and oxygen. In mammalian cells, CAT is primarily present in peroxisomes, and its absence in mitochondria is compensated by glutathione peroxidase [95,96].

### 6.2. Glutathione (GSH), Glutathione Peroxidase (GPx) and Glutathione Reductase (GRd)

Glutathione (GSH) is a tripeptide, possessing l-γ-glutamyl-l-cysteinyl-glycine structure, present in most cells. GSH is synthetized *de novo* in the cytosol through a two-step process starting from l-glutamate and cysteine to form γ-glutamylcysteine intermediate via the enzyme glutamate cysteine ligase (γ-glutamylcysteine synthetase). Subsequently, l-glycine is added to the C-terminus of γ-glutamylcysteine via the enzyme glutathione synthetase to form glutathione. GSH is maintained at high concentrations in cells, where it performs a role as an antioxidant. GSH serves as electron donor to H_2_O_2_ and lipid peroxides, that are reduced to water and lipid alcohols, respectively, in reactions catalysed by glutathione peroxidase (GPx) [97,98,99].

Glutathione peroxidase (GPx), present in the cytosol and in mitochondria, is a tetrameric enzyme containing seleno-cysteine (SeC) in the active site [99,100]. The SeC active site (Se-H) reacts with peroxide to form a selenenic acid (Se-OH), which is reduced by GSH molecule, resulted in the formation of a glutathiolated selenol (Se-SG) intermediate. Subsequently, the Se-SG bond is reduced by second GSH molecule, leading to the restoration of the GPx active site and the formation of oxidized glutathione (GSSG). GSSG, formed via creating a disulphide bridge between two glutathione molecules, is a product of GPx-catalysed reduction of peroxides [99]. GSSG can be reduced to GSH in reaction catalysed by glutathione reductase (GRd).

Glutathione reductase (GRd), a homodimer containing one flavine adenine dinucleotide (FAD) per subunit, catalyses the conversion of GSSG to GSH via using the reduced form of nicotinamide adenine dinucleotide phosphate (NADPH). Mechanism of GSH restoration from GSSG involves GPx reduction by NADPH and transfer of electrons to GSSG, facilitated by several key residues in GPx active site [101]. The high availability of GSH is important for scavenging oxidants and maintaining redox-state balance in healthy cells [98]. The intracellular concentrations of GSH can be increased via exogenous sources, such as N-acetylcysteine.

### 6.3. Thioredoxins

Thioredoxins (Trx) are proteins performing important role as endogenous antioxidant system against oxidative stress. The Trx antioxidant system is composed by thioredoxin (Trx), NADPH and thioredoxin reductase (TrxR). Trx, an enzyme with cys-gly-pro-cys in its active site, can be present in either oxidized disulphide form (Trx-S_2_) or reduced dithiol form [Trx-(SH)_2_]. The reduced form of Trx can act as a reductase towards disulphide bonds in oxidatively damaged proteins, via a disulphide–dithiol exchange mechanism resulting in thiol restoration in targeted protein and Trx oxidation [7,102]. The disulphides in oxidized Trxs are converted to dithiol form in reaction involving the presence of NADPH and catalysed by thioredoxin reductase (TrxR), a selenocysteine and FAD-containing protein [103,104]. The TrxR/Trx system can also catalyse reduction of dehydroascorbate [104] and reduction of GSSG to GSH [101].

Glutaredoxins (Grx) are intracellular redox enzymes belonging to the Trx protein family. Grx can catalyse reduction of disulphide bonds in substrates via dithiol and/or monothiol mechanism. In the first reaction mode, dithiols in Grx [Grx-(SH)_2_] reduce disulphide in target proteins via disulphide–dithiol exchange mechanism, with the formation of oxidized Grx (Grx-S_2_) [102]. The active site of oxidized Grx can be reduced back to dithiol form via GSH. In the monothiol reaction mode, Grx-SH catalyses the reduction of the disulphide bond (protein-S-SG) between GSH and target protein, resulting in the release of protein-SH and formation of a new disulphide (Grx-S-SG) between Grx and GSH. Subsequently, the Grx-S-SG disulphide is reduced by another GSH molecule, yielding Grx-SH and GSSG [105,106]. 

### 6.4. Other Antioxidant Proteins

Except for enzymes, there are other anti-oxidant proteins such as small proline-rich proteins and ferritin, that participate in anti-oxidant system in human body. 

Small proline-rich proteins (SPRRs) are structural components of cornified envelope in corneocytes, localized in the outermost layer of skin known as stratum corneum [107]. In the structure of cornified cell envelope (CE), SPRRs serve as crosslinking proteins via transglutaminase-mediated formation of *ε*-(*γ*-glutamyl) lysine cross-linkages with other proteins such as loricrin, thereby increasing the rigidity of CE [107,108,109]. The structure of SPRRs, composed of repeating β-turns, is determined by proline content, while high number of cysteine (-SH) residues in SPRRs are responsible for ROS quenching, with formation of inter- and intramolecular disulphide (S–S) bonds [110]. Therefore, SPRRs are part of defence mechanism protecting epidermis from ROS damage induced by UV, xenobiotics and pollutants [111].

Ferritin is a ubiquitous protein, distributed in the serum and in the cytoplasm, nucleus and mitochondria of cells, with a principal function for iron storage. The ferritin molecule consists of 24 subunits, organised within two distinct subunit types, heavy (H) and light (L), forming together a spherical structure [112,113,114]. Ferritin possesses antioxidant properties by sequestering iron, which in free form (Fe^2+^) can catalyse reduction of H_2_O_2_ and production of highly reactive •OH via Fenton chemistry [115]. The H subunit of ferritin possesses ferroxidase activity catalysing oxidation of Fe^2+^ to stable Fe^3+^, while the L subunit stabilizes protein structure and facilitates the uptake of iron, stored inside the ferritin shell. Even 4500 iron atoms can be sequestered by ferritin, in the balance between ferritin-bound iron (Fe^3+^) and Fe^2+^ pool in the cells, thereby preventing ROS generation via Fenton reaction [112,113,114]. It was suggested that increase in ferritin synthesis can serve as the defence mechanism against oxidative stress [116,117], and overexpression of ferritin H or L subunits diminished ROS formation in HeLa cells exposed to H_2_O_2_ [117].

### 6.5. Antioxidant Low Molecular Weight Molecules

Except for enzymes and other proteins, there are anti-oxidant low molecular weight molecules, of endogenous and exogenous origin, participating in anti-oxidant system in human body.

Coenzyme Q_10_ (CoQ_10_, ubiquinone), with a structure of 2,3-dimethoxy-5-methyl-6-decaprenyl-1,4-benzoquinone, is a lipophilic molecule synthetized in human and animal cells [118]. In CoQ_10_, a benzoquinone ring is synthetized from tyrosine, with 4-hydroxybenzoate (4HB) as a precursor of CoQ, while an isoprenoid side chain of CoQ_10_ is obtained from farnesyl pyrophosphate, a product of the mevalonate pathway [119]. CoQ_10_, as a component of the mitochondrial respiratory chain, accepts electrons from complex I (NADH: coenzyme Q reductase) and complex II (succinate: coenzyme Q reductase) and transfers (as CoQ_10_H_2_) electrons to complex III (coenzyme Q: cytochrome c reductase) [42]. The reduced form of CoQ_10_ (CoQ_10_H_2_, ubiquinol) acts as a phenolic antioxidant, protecting DNA, membrane phospholipids and mitochondrial membrane phospholipids from free-radical-induced oxidative damage [120]. Coenzyme Q_10_ can be also obtained exogenously via supplementation. A meta-analysis comprising multiple clinical trials concluded that CoQ_10_ administration dosages resulted in the decrease in MDA levels amongst participants [121].

Melatonin, also named as N-acetyl-5-methoxy-tryptamine, is an endogenous indole hormone controlling physiologic processes such as sleep and circadian rhythm [122]. Melatonin is secreted by the pineal gland, where it is synthetized via a pathway involving tryptophane and serotonin, but its synthesis also occurs in brain, lens, skin, retina, lymphocytes and bone marrow [123]. Melatonin is synthetized, taken up by, and concentrated in mitochondria [122]. Melatonin functions as an antioxidant by directly scavenging free radicals, stimulating anti-oxidant enzymes (SOD, CAT, GPx, GRd), as well as improving mitochondrial OXPHOS efficiency [124]. Melatonin is a direct ROS scavenger (^1^O_2_, O_2_^•^^−^,•OH) by donating electron(s) to the ROS, with the formation of products such as cyclic 3-hydroxymelatonin or indolyl radical cation of low reactivity [123]. Melatonin can be also supplied exogenously from diet or supplements [125] to heal jet lag, insomnia, narcolepsy and other sleep disorders, and is considered as a potential cardioprotective, anti-inflammatory and anti-cancer agent [126].

Bilirubin (BR) is a tetrapyrrole pigment, composed of two rigid dipyrroles joined by a methylene bridge at carbon 10, present in the plasma as a form bound to albumin, while a form conjugated with glucuronic acid is excreted into the intestine with bile [127,128]. Synthesis of bilirubin includes two steps, the cleavage of heme IX via heme-oxygenase (HO) into biliverdin IX*α*, carbon monoxide and free ferrous iron, and subsequent conversion of biliverdin (BV) into bilirubin IX*α* via biliverdin reductase [128,129]. Bilirubin is a strong endogenous anti-oxidant cytoprotectant that interacts with free oxygen radicals resulting in the oxidation of BR to BV and immediate reduction to bilirubin via biliverdin reductase [130]. Bilirubin was reported to neutralize free radicals, providing efficient protection against 10,000-fold higher concentration of H_2_O_2_ [128,130], and prevent peroxidation of lipids [131]. The anti-oxidant properties of bilirubin also stem from its ability to inhibit NADPH oxidase [132]. Bilirubin is considered as a crucial substance acting as an antioxidant substance in serum of human beings [131].

α-Lipoic acid (α-LA), also named as 1,2-dithiolane-3-pentanoic acid, is an organosulfur compound synthetized by plants, animals and humans [133]. α-LA is synthetized in mitochondria from octanoic acid and cysteine, and play a crucial role in mitochondrial bioenergetic reactions. α-LA can be reduced by NADH/NADPH to dihydrolipoic acid (DHLA), and both forms possess anti-oxidant activity. α-LA and DHLA are capable of scavenging hydroxyl radicals and preventing protein carbonyl formation, whereas DHLA can also reduce the oxidized forms of vitamin C and E, and GSH [134]. α-LA is synthetized endogenously, but is also considered as a valuable supplement with beneficial therapeutic effects [135,136,137].

N-acetylcysteine (NAC), a derivative of amino acid l-cysteine, is used as a drug for the treatment of acetaminophen overdose and as a mucolytic agent in respiratory diseases. NAC, administered orally, intravenously or by inhalation, is metabolized into cysteine, cystine, inorganic sulfate and glutathione [138]. N-acetylcysteine possesses direct and indirect antioxidant activity. In a direct mode, sulfhydryl group (-SH) in the NAC reduces radical species via electron donation. In an indirect antioxidant action, NAC undergoes deacetylation via acylase to cysteine, the building block in glutathione synthesis. NAC can also break S-S bonds via thiol-disulphide interchange reaction, thereby restoring thiol pools involved in redox state regulation [139]. NAC can also bind to metal ions, such as copper (Cu^2+^), iron (Fe^3+^), cadmium (Cd^2+^), mercury (Hg^2+^) and lead (Pb^2+^), via forming complexes which are excreted from the body [138]. N-acetylcysteine is considered as a valuable supplement for conventional treatment of oxidative stress-linked diseases [140].

Resveratrol (RV) is a plant-derived stilbene polyphenol (3,5,4′-trihydroxystilbene) compound, found in numerous food sources including grapes, wine, blueberry, bilberry, cranberry and peanuts [141]. RV is administered orally, absorbed via passive diffusion or in complex with membrane transporters and released into the bloodstream. In liver, RV is converted to conjugated glucuronides and sulfate metabolites possessing biological activity [142]. Resveratrol possesses a wide range of biological properties, such as cardioprotective, neuroprotective, anti-inflammatory, anticancer, antidiabetic, antimicrobial and antioxidant activities [143]. Resveratrol showed hydrogen peroxide and superoxide radical scavenging activities, as well as ferrous ion (Fe^2+^) chelating activity [144]. The ROS-scavenging activity of resveratrol depends on hydrogen donation via hydrogen abstraction from *para*-OH (4′-OH) group or from *meta*-OH (3-OH or 5-OH) group, leading to generation of resveratrol phenoxyl radicals, which structures can undergo further reorganization to form semiquinones [145]. Resveratrol can chelate the ferrous ion with its two hydroxyl groups (3-OH and 5-OH), forming a complex composed of one Fe^2+^ and two resveratrol molecules [144]. Resveratrol also possesses indirect antioxidant activity as a gene regulator. Resveratrol was reported to increase the expression of antioxidant enzymes (SOD, GPx, CAT), down-regulate the expression and activity of NADPH oxidase, and reduce mitochondrial superoxide generation via stimulating mitochondria biogenesis [146].

Vitamins A, C and E are important constituents of anti-oxidant barrier against oxidative stress in human body.

## 7. Vitamins as Antioxidants

β-Carotene (as precursor of vitamin A), tocopherols (as vitamin E forms) and ascorbate (vitamin C) are described in terms of their structures and anti-oxidant activities.

### 7.1. Structural and Antioxidant Characteristics of β-Carotene, Tocopherols and Ascorbate

#### 7.1.1. β-Carotene: Structure and Anti-Oxidant Property

**Figure 2:**
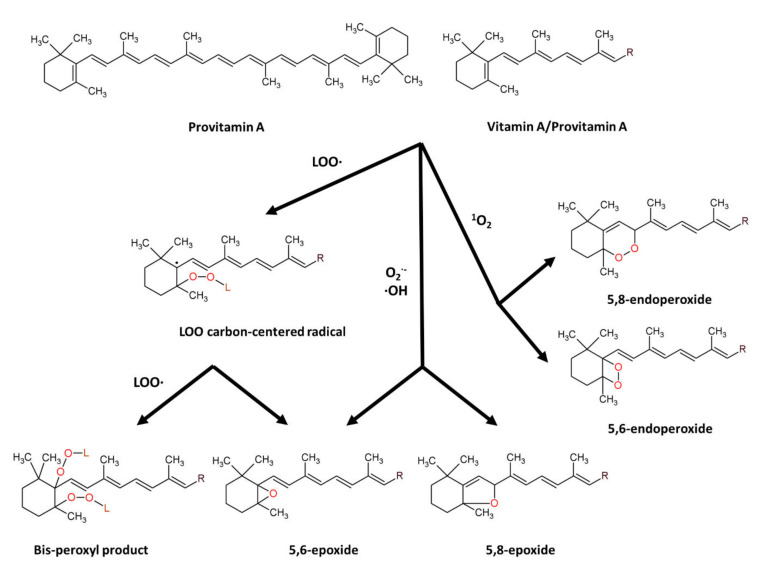
The scavenging mechanism of provitamin A and vitamin A.

β-carotene (β-C) is a non-oxygenated carotenoid molecule, with 40 carbons, 11 conjugated double bonds and 2 β-ionone rings (Figure 2), possessing provitamin A activity [147,148]. β-carotene is an antioxidant molecule serving as a quencher of singlet oxygen and a scavenger of peroxyl radicals. The physical quenching of singlet oxygen is carried out by transferring excitation energy from singlet oxygen to carotenoid molecule, to generate excited triplet-state β-carotene and ground-state oxygen. Subsequently, the energy is dissipated between the excited β-C and the surroundings to yield normal energy state carotenoid and thermal energy [147,148]. The chemical quenching of singlet oxygen is carried out by oxygenation of β-carotene with the formation of β-C endoperoxides [149,150]. The scavenging of peroxyl radicals is carried out by addition of peroxyl radical to a suitable double bond in provitamin A/vitamin A molecules resulting in formation of carbon radical that can further undergo the conversion into epoxides or react with new peroxyl radical to form bis-peroxyl products [71,151,152]. β-carotene also showed scavenging activity towards superoxide anion radical, hydroxyl radical and hydrogen peroxide [153], and reaction of carotenoids with superoxide anion or hydroxyl radical results in formation of carotenoid epoxides [154,155].

#### 7.1.2. Tocopherols: Structure and Anti-Oxidant Property

**Figure 3:**
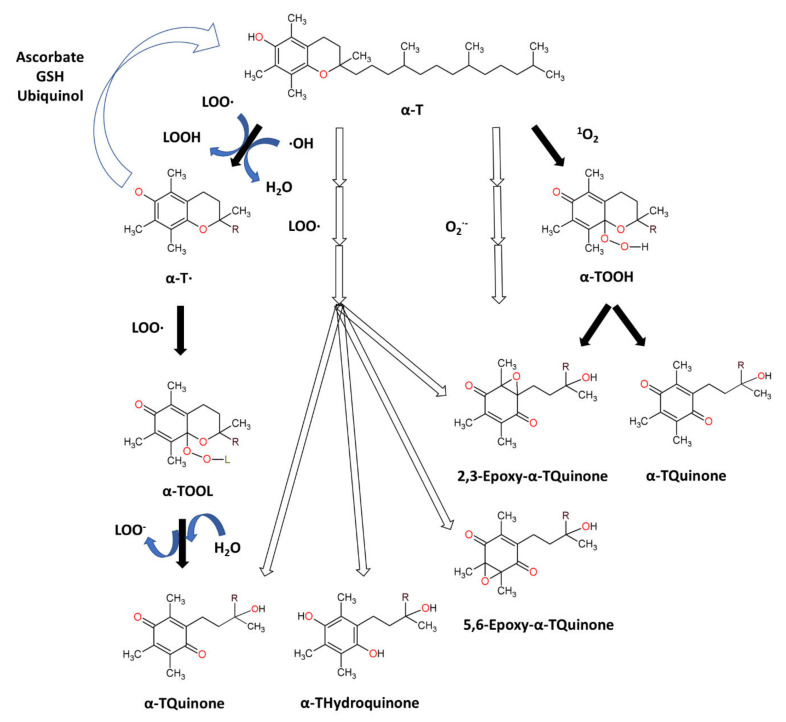
The scavenging mechanism of α-tocopherol.

Tocopherols are plant-derived lipid-soluble molecules, possessing a chromanol ring and a saturated 16-carbon atom phytyl chain, that belong together with tocotrienols to the group of vitamin E. There are four isoforms of tocopherol (α-, β-, γ-, δ-), differing in the number of CH_3_− group substitutions on chromanol ring. Tocopherols can be distinguished from tocotrienols, which possess an unsaturated phytyl (farnesyl) chain. α-Tocopherol (α-T) (Figure 3) is an antioxidant that functions as a “chain breaker” during lipid peroxidation in cell membranes and various lipid particles [71]. α-T terminates the lipid peroxidation chain reactions by donating its phenolic hydrogen atom to a lipid peroxyl radical (LOO•), leading to the formation of lipid hydroperoxide (LOOH) and α-tocopheroxyl radical [71,156]. The formed tocopheroxyl radical, insufficiently reactive to initiate lipid peroxidation, can react with another lipid peroxyl radical (LOO•) to yield a non-radical product α-tocopherylquinone [152,156]. The tocopheroxyl radical can be also regenerated to tocopherol via reduction by ascorbate and glutathione [71] or ubiquinol [157]. α-T can also react with hydroxyl radical to form the tocopheroxy radical [158], and react with superoxide [159] or singlet oxygen [160] to form α-tocopheryl quinones, including α-tocopheryl quinone epoxide [161,162]. 

#### 7.1.3. Ascorbic Acid: Structure and Anti-Oxidant Property

**Figure 4:**
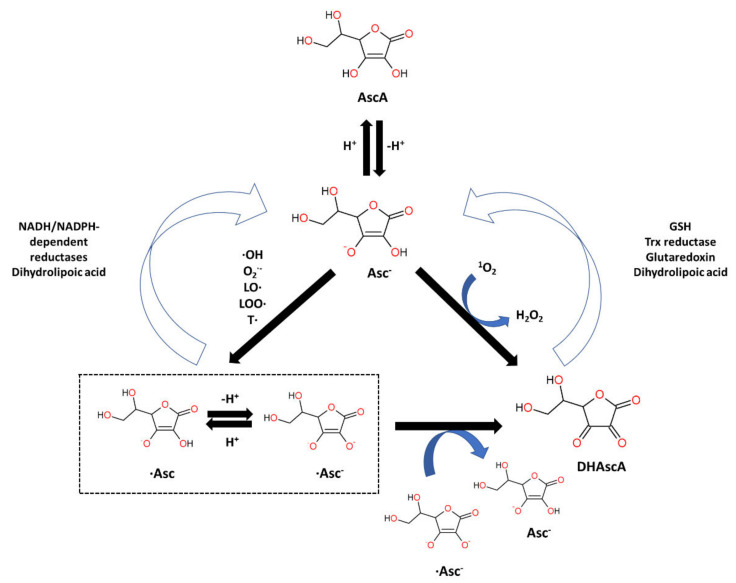
The scavenging mechanism of ascorbate.

Ascorbic acid (AscA), also called vitamin C, is a water-soluble ketolactone with two ionizable hydroxyl groups. AscA exists in a form of monoanion ascorbate or can undergo oxidation to dehydroascorbic acid (DHAscA) [163,164]. Ascorbic acid is a potent antioxidant by scavenging oxygen radicals (Figure 4). Ascorbate, a prominent form of vitamin C at physiological pH, reduces radicals (superoxide, hydroxyl radical, alkoxyl radical, peroxyl radicals, tocopheroxyl radicals) by donating an electron, and in turn forming the ascorbate radical [163]. Ascorbate can also donate two electrons to singlet oxygen, resulting in the formation of DHAscA and H_2_O_2_ [165]. Ascorbate radical can undergo reaction with another ascorbate radical to yield ascorbate and DHAscA [71]. Ascorbate can be also regenerated from ascorbate radical via NADH/NADPH-dependent reductases, and from DHAscA by glutathione, thioredoxin reductase and glutaredoxin [163,166]. Dihydrolipoic acid is capable of reducing dehydroascorbic acid to ascorbate, and can also reduce ascorbate radical [167].

### 7.2. In Vivo and In Vitro Antioxidant Characteristics of β-Carotene, Tocopherols and Ascorbate

#### 7.2.1. Oxidative Stress Inducers Used during In Vitro and In Vivo Studies

**Figure 5:**
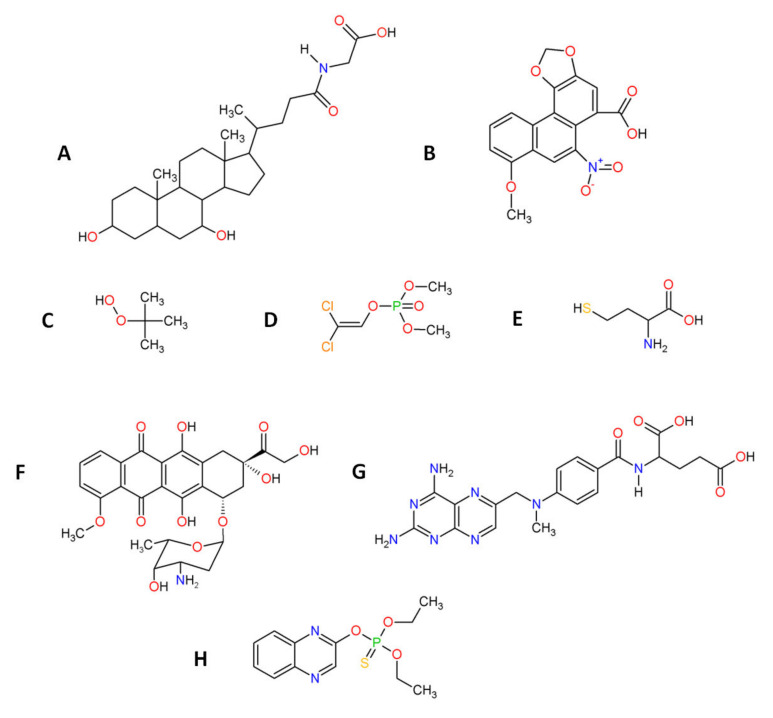
Exemplary organic stress inducers used during in vitro and/or in vivo tests. Structures of glycochenodeoxycholic acid (**A**), aristolochic acid (**B**), t-BuOOH (**C**), dichlorvos (**D**), homocysteine (**E**), doxorubicin (**F**), methotrexate (**G**) and quinalphos (**H**).

Artificial stress inducers are conditions or compounds applied in order to induce oxidative stress in cells, tissues or organisms. Stress inducers are used to study the counter, anti-oxidant effect of β-carotene, tocopherols and ascorbic acid during in vitro and in vivo studies. For in vitro tests, conditions such as irradiation (UVA, UVB) and molecules such as H_2_O_2_ are used to procure oxidative stress in target cells. Except for H_2_O_2_, other inorganic and organic molecules are used during anti-oxidant studies of β-carotene, tocopherols and ascorbic acid. These compounds possess different chemical structures (Figure 5) and induce oxidative stress in cells/organisms via different mechanisms. Tert-butyl hydroperoxide (t-BuOOH) can be metabolized by cytochrome P450 with generation of peroxyl and alkoxyl radicals, or can be converted by the glutathione system to tert-butyl alcohol and GSSG, causing glutathione (GSH) depletion [168]. Glycochenodeoxycholic acid (GCDCA) increases mitochondrial oxidative stress through the up-regulation of acetylated SOD2 and inhibition of SOD2 activity [169]. Aristolochic acid (AA) exerts oxidative stress via depleting glutathione pool, and also induces genotoxicity via formation of AA-derived DNA adducts or possibly by down-regulating the expression of DNA repair genes [170]. Dichlorvos affect metabolism through inhibiting carboxyl ester hydrolases, causing DNA alkylation and interfering with mitochondrial bioenergetics [171]. Homocysteine (Hcy) can affect metabolism via inhibiting Na^+^/K^+^-ATPase, and can also generate oxidative stress through auto-oxidation [172]. Sodium selenite can be converted by the glutathione system to selenium, with ROS generation [168]. Advanced glycation end products (AGEs) bind to RAGE receptor initiating a range of downstream effects such as the activation of NADPH oxidase [173]. Ethanol is converted by alcohol dehydrogenase to acetaldehyde, which subsequently oxidized via aldehyde dehydrogenase to acetate, the latter possessing toxic activity [168]. For in vivo test, conditions such as heat stress and surgery injury and compounds such as heavy metals, doxorubicin, sodium azide and quinalphos were used to evaluate anti-oxidant activity of β-carotene, tocopherols and ascorbate. Cadmium (Cd^2+^) presence can decrease glutathione pool and Cd^2+^ can also bind to protein thiols in mitochondrial membrane, thereby affecting mitochondrial permeability transition, and can inhibit mitochondrial complex III, resulting in accumulation of semiubiquinones and superoxide anion generation [174]. Sodium azide (NaN_3_) inhibits the activity of mitochondrial cytochrome oxidase resulting in ROS overproduction [175]. Doxorubicin (DOX) is an anthracycline that within mitochondria undergoes conversion to semiquinone via one-electron reduction of the quinone moiety, and reacts with oxygen to generate superoxide anion [176]. Methotrexate (MTX) toxicity is due to inhibition of DNA synthesis [177], reduction in methionine synthesis, decrease of S-Adenosyl Methionine and affecting methylation reactions [177,178]. MTX also inhibits NADP-dehydrogenases and NADP malic enzyme [178], leading to the decreased availability of NADPH used by glutathione reductase, consequently resulting in the diminished pool of reduced glutathione (GSH) [179]. Organophosphate pesticides, such as dichlorvos and quinalphos, inhibit activity of acetylcholinesterase (AChE) leading to accumulation of acetylcholine [180]. β-cyfluthrin/cyfluthrin are synthetic fluorinated pyrethroid insecticides that alter the permeability of sodium channels, inhibit calcium transport enzymes and also generate ROS via quick metabolism of synthetic pyrethroids [181,182].

#### 7.2.2. β-Carotene: Antioxidant Activity In Vitro

β-carotene has been tested in numerous in vitro studies in terms of its ability to prevent or diminish oxidative stress in human or animal cells (Table 2). In one study, β-carotene was reported to reduce ROS accumulation in undifferentiated rat pheochromocytoma (PC12) cells exposed to H_2_O_2_ [153]. In another study, β-C pre-treatment of rat hepatocytes reduced the level of oxidative stress induced as a result of hepatocyte exposure to glycochenodeoxycholic acid [183]. Furthermore, β-carotene was reported to reduce the level of ROS produced in the oocytes upon Rosup exposure [184]. Furthermore, treatment of human erythrocytes with β-carotene prevented the increase in lipid peroxidation caused by exposure to H_2_O_2_. Moreover, the activities of SOD and CAT in H_2_O_2_-exposed erythrocytes were restored in the presence of β-C [185]. Moreover, pre-incubation of mice-derived erythrocytes with β-carotene reduced the level of MDA, increased during erythrocytes incubation in the presence of H_2_O_2_ [186]. β-carotene also suppressed oxidative stress in cardiomyocyte cells (H9c2), induced as a consequence of advanced glycation end (AGEs) products exposure. β-C treatment decreased ROS production and MDA content, and restored GPx and SOD activity in AGEs-treated H9c2 cells [187]. Murine normal and tumour thymocytes cultivated in the presence of t-BuOOH showed increased MDA content. Enrichment of cells with β-C alleviated the t-BuOOH-induced MDA increase during cultivation in both cell types [188].

#### 7.2.3. Tocopherols: Antioxidant Activity In Vitro

The ability of tocopherols to decrease oxidative stress has been proved in numerous studies (Table 3). α-T showed hydrogen peroxide and superoxide radical scavenging activities, as well as ferrous ion (Fe^2+^) chelating activity [144]. Pre-treatment of human keratinocyte cells with α-T decreased the level of MDA and ROS, induced due to keratinocyte exposure to ultraviolet A radiation [189]. α-T suppressed formation of lipid peroxidation and protein carbonyl products in human neuroblastoma (SH-SY5Y) cells exposed to advanced glycation end products (AGEs) [173]. In another study, vitamin E supplementation decreased ROS and MDA levels promoted in human umbilical vein endothelial cells after exposure to homocysteine (Hcy) [172]. Treatment of human erythrocytes with α-T diminished the increase in MDA content exerted by pesticide dichlorvos [190]. Furthermore, treatment of human colorectal adenocarcinoma cell line (Caco-2) with vitamin E reduced the level of MDA, increased in Caco-2 due to exposure to H_2_O_2_ [191]. Furthermore, pre-treatment of rat hepatocytes with α-T reduced the level of oxidative stress induced as a result of hepatocyte exposure to glycochenodeoxycholic acid [183]. α-T also attenuated the H_2_O_2_ level in rat renal tubular epithelial cells (NRK-52E), increased in NRK-52E cells due to exposure to aristolochic acid [192].

#### 7.2.4. Ascorbic Acid: Antioxidant Activity In Vitro

Antioxidant activity of vitamin C has been confirmed in different reports (Table 4). In one study, pre-treatment of human embryonic kidney (HEK293) cells with ascorbic acid caused the decrease in levels of ROS production, MDA, protein carbonyl and 8-hydroxy-2-deoxy guanosine (8-OHdG), accumulated in HEK293 cells due to exposure to H_2_O_2_ [193]. In another study, pre-treatment of human lens epithelial cells (LEC) with vitamin C caused the reduction in ROS activity, increased in H_2_O_2_-exposed LEC cells [194]. In another study, ascorbate effectively reduced ROS level in human retinal pigment epithelial (ARPE-19) cells, increased as a result of cells exposure to H_2_O_2_ or UVB irradiation [195]. Moreover, treatment of human erythrocytes with ascorbic acid diminished the increase in MDA content induced by pesticide dichlorvos [190]. Moreover, Vit C also reduced the H_2_O_2_ level in rat renal tubular epithelial cells (NRK-52E), increased in NRK-52E cells due to exposure to aristolochic acid [196]. Furthermore, the presence of ascorbic acid in human hepatoma (HepG2) cells alleviated MDA induction, caused by exposure of HepG2 cells to ethanol, sodium selenite or t-BuOOH. Ascorbic acid presence also caused the restoration in SOD activity, CAT activity and GSH content in oxidative stress-induced HepG2 cells [168]. In one more study, UV radiation or H_2_O_2_ exposure induced oxidative stress (ROS, MDA, 4-HNE, Isoprostanes) in human skin fibroblasts (CCD 1112Sk), and incubation with ascorbic acid led to reduction in OS level in CCD 1112Sk cells [197].

#### 7.2.5. β-Carotene: Antioxidant Activity In Vivo

**Figure 6:**
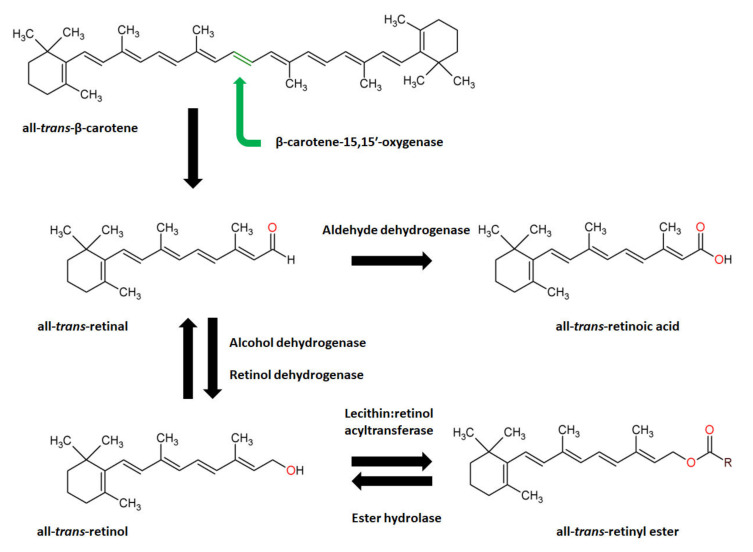
The structures of provitamin A (β-carotene) and vitamin A constituents (retinaldehyde, retinol, retinoic acid).

Carotenoids, such as β-carotene, are lipid soluble tetraterpenoid molecules naturally found in plants and microorganisms (algae, yeast and some bacteria), and available in food. In mammals, lipid-soluble β-carotene is absorbed in small intestine and delivered to the peripheral tissues (liver, adipose tissue, kidney, skin, lungs) via various lipoprotein particles. In one of the possible mechanisms for vitamin A synthesis (Figure 6), all *trans* β-carotene, the predominant form of β-carotene found in nature, is symmetrically cleaved by the enzyme β-carotene-15,15′-oxygenase (CMOI) to yield two molecules of retinaldehyde. Ingested β-C can be cleaved via CMOI in intestine and in various tissues within the body. Retinaldehyde can be reduced via alcohol dehydrogenase or retinol dehydrogenase to retinol, a vitamin A. Retinol can be also esterified via lecithin:retinol acyltransferase (LRAT) to retinyl esters, which constitute vitamin A reserves. Retinaldehyde can be also oxidized, by enzymes (ALDH 1 or RALDH) from the aldehyde dehydrogenase 1 family, into all-*trans* retinoic acid which constitutes the biologically active form of vitamin A, responsible for transcriptional regulation [198,199]. 

The antioxidant activity of β-carotene was evaluated in vivo with different animal species (Table 5). Wistar rats were characterized by increased MDA level and decreased SOD and CAT activity, as a result of 2-week high-fat diet (HFD), if compared to control where normal diet was administered. The addition of β-carotene for two weeks, before or after 12-week HFD, resulted in reduction of MDA level and restoration in SOD and CAT activity, in investigated rats [200]. Wistar albino rats were administered methotrexate (MTX) what resulted in the increased MDA level and reduced activities of SOD, CAT and GPx in the livers of rats exposed to MTX. Co-administration of MTX with β-carotene diminished hepatic MDA level and restored activities of SOD, CAT and GPx in rat livers [201]. β-carotene was reported to attenuate oxidative stress in the spinal cord of rats with spinal cord injury (SCI). Decreased ROS production and MDA level, and restored SOD activity were detected in spinal cord tissues of SCI rats fed with β-C [202]. β-carotene also diminished oxidative stress in mice with traumatic brain injury (TBI). Decreased MDA content and restored SOD activity were detected in brain tissue of TBI-mice, when β-C doses were administered [203]. In one more study, incubation of *Drosophila melanogaster* larvae with β-carotene diminished the OS level induced as a consequence of larvae exposure to γ-irradiation [204].

#### 7.2.6. Tocopherols: Antioxidant Activity In Vivo

Vitamin E is found in vegetable oils and seeds, with α-tocopherol being commonly available in wheat germ, olive, and sunflower oil, and γ-tocopherol being prominent in soybean, corn, and cottonseed oil. Lipid-containing food intake is the source of vitamin E in human body [157,205,206]. During the digestion process, triacylglycerols and other esterified fat-soluble compounds are partially processed in the stomach by gastric lipase [207], and absorption of vitamin E occurs in the small intestine [157]. Vitamin E along with other lipids is incorporated into mixed micelles, the process aided by pancreatic lipases and bile salts, in the duodenum [206,207]. In the intestine lumen, micelles solubilize hydrophobic components and diffuse into the glycocalix to approach the brush border membrane of the enterocytes [157,208]. Vitamin E transport across the enterocyte membrane occurs via passive diffusion and/or is also mediated by membrane proteins (NPC1L1, SR-BI, CD36) [208]. In enterocytes, vitamin E is incorporated into chylomicrons, which are secreted into the intestinal lymph system and released into the bloodstream [208,209]. Vitamin E, transported in blood via different lipoproteins, is distributed within liver and extrahepatic tissues (adipose tissue, muscle, adrenal glands) [206,209]. In liver, RRR α-tocopherol selectively binds to the α-tocopherol transfer protein (α-TTP), while other isoforms of vitamin E are excreted in the bile [206]. α-Tocopherol, incorporated via α-TTP into lipoproteins and re-secreted to the circulation [206], is the major tocopherol found in human blood and tissues [205].

The antioxidant effect of α-tocopherol has been tested in vivo on animal species (Table 5). A 15-day intake of α-T by Wistar rats led to reduction in lipoperoxide concentrations, increased as a consequence of intraperitoneal injection of Cd^2+^ ions [210]. In another study, vitamin E administration reduced cardiac MDA level in rats, increased due to doxorubicin (DOX) injection. Additionally, cardiac GSH level in DOX-injected rats fed with vitamin E were higher, if compared to DOX-injected group without vitamin administered [211]. Further, administration of Vit E (α-tocopheryl acetate) to female white mice treated with cyfluthrin resulted in the reduction in plasma MDA level and elevation in erythrocytes CAT activity, if compared to group where cyfluthrin was administered, but without Vit E [181]. In another study, α-T decreased ischemia/reperfusion injury-induced ROS production and oxidative modification of phospholipids in mice [212]. Vitamin E was also tested in terms of its cytoprotective effect towards cardiovascular system in mice subjected to heat stress. Heart tissue of mice exposed to heat stress (HS) conditions showed increased ROS levels, but administration of vitamin E possessed ameliorating effect. Moreover, cardiomyocytes of mice subjected to heat stress and fed with vitamin E showed decreased MDA levels and restored SOD and GSH levels, if compared to HS conditions but without vitamin administration [213]. Supplementation of VitE (α-tocopheryl acetate) to rat diet reduced plasma lipid peroxidation and restored plasma SOD and GPx activity, affected due to rats feeding on high-fat diet [214]. Moreover, supplementation of VitE (α-tocopheryl acetate) reduced MDA serum concentration and MDA breast muscle content in chicken broilers, fed on linseed oil-enriched diet [215].

#### 7.2.7. Ascorbic Acid: Antioxidant Activity In Vivo

Vitamin C is naturally present in fruits (kiwifruit, orange, lemon, black currant, raspberry, strawberry, grapes etc.) and vegetables (broccoli, cabbage, spinach, tomato, potato, pepper etc.) [216,217], but can be also manufactured industrially in reactions involving various intermediates such as d-sorbitol, l-sorbose and 2-keto-l-gulonic acid [218]. In animals, l-ascorbic acid is synthetized through glucuronic acid pathway in liver (mammals) or kidney (reptiles, birds) [219]. Humans, apes, guinea pigs and fruit-eating bats cannot synthetize ascorbic acid due to the lack of enzyme l-gulonolactone oxidase [220]. In humans, ascorbate and DHAscA (vitamin C equivalents) are absorbed from ingested food by enterocytes of the small intestine. Ascorbate is absorbed via Na^+^-dependent vitamin C transporters (SVCTs) while DHAscA is absorbed via Na^+^-independent facilitative glucose transporters (GLUTs) and reduced to ascorbate. Vitamin C, absorbed from the intestinal lumen, is transported with the blood to various peripheral organs that differ in tissue ascorbate content [163,164,221]. 

The antioxidant effect of ascorbic acid has been tested in vivo on animal species (Table 5). In a study comprising Wistar rats, a 9-day supplementation of ascorbic acid resulted in the decreased levels of malondialdehyde (MDA) and protein carbonyl, increased in the stomach, colon and kidneys of rats feeding with sodium azide (NaN_3_), as an oxidative stress inducer [222]. In another study, the antioxidant activity of ascorbate was tested in doxorubicin-administered male Wistar rats. The cardiac tissue of DOX-administered rats fed with ascorbate, showed reduced levels of superoxide anion, lipid peroxidation and protein carbonyl products, if compared to the group subjected to DOX administration but without Vit C treatment. Moreover, ascorbate treatment restored SOD and GPx activities, decreased in rat cardiac tissue due to DOX administration [223]. In another study, insecticide quinalphos (QP) administered to Sprague–Dawley rats caused induction of oxidative stress in rat cardiac tissue, indicated by reduced GPx activity and CAT activity, and increased MDA content. Co-administration of Vit C to investigated rat group resulted in the alleviation of oxidative stress, expressed by restored GPx activity and CAT activity, and reduced MDA content in QP-exposed rat cardiac tissue [224].

#### 7.2.8. Antioxidant Characteristics of β-Carotene, Tocopherols and Ascorbate Based on Populational/Clinical Human Studies

The characteristics of β-carotene, tocopherols and ascorbate were also characterized in terms of their antioxidant activities in human studies (Table 6). Antioxidant activities of β-carotene were proved in exemplary populational studies. In one study, including 85 healthy male 22–58 age-volunteers exposed to Pb for 4 to 38 years, the anti-oxidant effect of β-carotene was evaluated. The oral administration of β-carotene (10 mg/day) to 35 participants for 3 months, resulted in reduced concentration of lipid hydroperoxides (LHP) in serum and decreased malondialdehyde (MDA) level in erythrocytes and leukocytes, if compared to a group (50 participant) without β-C administered [225]. In another study, a 3 week-supplementation of dietary (9-*cis*, all-*trans*) β-carotene (60 mg/day) to patients (n = 20) with long-standing non-insulin-dependent diabetes mellitus (NIDDM) showed that low density lipoproteins of β-C administered NIDDM-patients possessed increased resistance to oxidation and reduced level of MDA [226]. Antioxidant activities of tocopherol and ascorbate were also proved in some populational studies. In a study with participants (n = 35) with polygenic hypercholesterolemia and enhanced oxidative stress, the 16-week administration of natural α-tocopherol (1600–3200 I.U.) to participants resulted in a observed reduction of plasma F_2_-isoprostane concentrations [227]. In a study, including 8 healthy white volunteers whose skin was exposed for 6 h to UV radiation, the 8-week supplementation with α-tocopherol (400 IU/day) caused the reduction in tissue MDA content, induced as a consequence of skin exposure to UVR [228]. Moreover in the study, comprising totally 704 participants at age of 77, the dietary intake of ascorbic acid and β-carotene resulted in the reduced urinary level of F_2_-isoprostanes, the biomarker of ROS-mediated peroxidation of arachidonic acid [229].

In a study consisting of 34 female participants with type 2 diabetes, the oral supplementation of α-tocopherol (800 IU/day) to 13 participants for 6 weeks resulted in the decrease in MDA erythrocyte level, if compared to a group (n = 21) without α-tocopherol administration [230]. In another study, 5 participants with diabetes mellitus type 2 who received vitamin C (1000 mg/day) for 6 weeks, showed decreased plasma levels of MDA and F_2_-isoprostanes, if compared to results before supplementation [231]. In a group of 40 participants with clinically diagnosed DMT2, hypoglycemic drug-administered diabetic (27 macrovascular, 13 microvascular) patients supplemented for 3 months with vitamin E (400 mg/day) showed decreased MDA serum concentration and increased serum SOD activity and erythrocyte GSH content, if compared to the beginning of study, or when compared with 40 DMT2 patients treated for 3 months with hypoglycemic drugs but without VitE supplementation [232]. The antioxidant activity of VitE has been also evaluated in a study comprising 72 patients with late-stage knee osteoarthritis (OA) and concluding that a two-month intake of VitE (400 IU) could reduce oxidative stress conditions (MDA in plasma and synovial fluid) in patients with OA [233].

#### 7.2.9. Antioxidant Characteristics of β-Carotene, Tocopherols and Ascorbate: A Summary

The literature data gathered show that β-carotene, tocopherols and ascorbic acid act as efficient antioxidants towards human and animal cells in vitro, under stress conditions. Supplementation with β-carotene, tocopherols or ascorbic acid, as free-radical quenchers, decreased the level of OS (bio)markers (MDA, ROS, Protein carbonyls, Isoprostanes, 8-OHdG) in numerous types of cells exposed to stress conditions. Conducted in vitro studies varied in terms of not only tested cell lines, but also research conditions including methods of oxidative stress induction, the form and concentration of antioxidant and the order of culture supplementation. These differences amongst studies can create limitations for results comparison and interpretations. Different methods, such as physicochemical factors as well as inorganic and organic oxidizing agents, were used to induce oxidative stress (OA) in investigated cell cultures. These stress inducers can cause oxidative stress via different mechanisms, and were tested towards different human and animal cells. Moreover, different stress inducers used for the same cell line can result in the different OS extent [186]. The concentrations of in vitro tested antioxidants differ amongst studies (Table 2, Table 3 and Table 4), but the anti-oxidant concentrations strictly influences the mitigation of oxidative stress [190]. Another important barrier for interpretation of anti-oxidative efficiency is the chemical form of anti-oxidant tested. Amongst tocopherols, α-T is commonly used during in vitro tests and its antioxidant activity was confirmed. However, the ability of α-T again to mitigate OS is not always proved and different tocopherol forms (α-T, γ-T) can possess different anti-oxidant activity [234]. Furthermore, antioxidant evaluation in vitro includes their different modes: cell pre-treatment with a selected antioxidant prior to oxidizing agent (OA)-exposure, co-treatment of cells with antioxidant and OA, or the subject of cells to oxidizing agent before adding the selected antioxidant. The differences in oxidizing agent/anti-oxidant supplementation order can also have influence of the outcome of research studies. Nevertheless, β-carotene, tocopherols and ascorbic acid proved to act as efficient antioxidants, despite differences between conducted studies. 

Animal in vivo studies (Table 5) show that β-carotene, tocopherols and ascorbic acid can be efficient compounds to mitigate oxidative stress, as indicated by decreased levels of OS biomarkers (MDA, Lipid peroxidation, Protein carbonyls) in investigated species. As in case of in vitro research, the problems with interpretation of different experimental conditions (OS generation method, supplementation mode) also occur, and further discrepancies (single cells vs. whole organism) between in vitro and in vivo appear. Nevertheless, the antioxidant properties of β-carotene, tocopherols and ascorbic acid was shown, despite differences between conducted in vivo studies. 

Some human populational/clinical studies (Table 6) show that β-carotene, α-tocopherol or ascorbic acid can improve anti-oxidant status in a selected group of participants. However, due to a scarce number of studies and a variety of conditions (different number of participants and health condition status, various time span and biomarkers) influencing studies outcome, the effectiveness of β-carotene, α-tocopherol or ascorbic acid as effective anti-oxidants, is not certain. Moreover, other reports do not confirm improvements in anti-oxidant level upon β-carotene [235], vitamin E [236] or ascorbic acid [237] administration. Moreover, the connection between free-radical scavenging ability of anti-oxidants and the use of antioxidants for prevention of human diseases has not been established. Although α-tocopherol exhibits anti-oxidant activity and inhibits oxidation of low-density lipoprotein cholesterol, α-T supplements failed to reduce atherosclerosis-related events [78]. What is more, beneficial effect of vitamin C in reference to human diseases (cancer, atherosclerosis, diabetes, neurodegenerative disease) remains equivocal [217], and a shortage of clinical trials impedes drawing clear conclusions about therapeutic role of ascorbic acid administration [238]. Furthermore, it was also reported that supplementation with anti-oxidant nutrients (vitamin C, vitamin E or β-carotene) offered no prevention against cancer incidence [239], and there is no certainty that possible cancer-preventive ability of anti-oxidant molecules, such as β-carotene, can be strictly due to free radical quenching mechanism [240]. In addition to mentioned discrepancies, it should be also noted that β-carotene and vitamin C can possess pro-oxidant activity [217,240]. Therefore, further studies are required to determine the effect of β-carotene, α-tocopherol or ascorbic acid on the level of oxidative stress and the antioxidant status in broader groups within healthy and disease-diagnosed populations.

Exposure to oxidative stress causes the alteration in anti-oxidant mechanisms, with the change in the activity of SOD, CAT and/or GPx (Table 2, Table 3, Table 4, Table 5 and Table 6). The exposure of cells to different OS inducers resulted in decrease in SOD, CAT and GPx activity. SOD, CAT and GPx are enzymes participating together to prevent oxidative damage to cells. Saturation of SOD and CAT during ROS conversion, and decreased availability of GSH for GPx can lead to decreased activities of these enzymes [190]. Reduced GPx activity can result in decreased SOD and CAT activities [179], and activity of SOD and CAT can be also inhibited by ROS, such as H_2_O_2_, [181] via affecting enzyme active site or via mechanism of cell regulation (de-/phosphorylation) [185]. Moreover, oxidizing agent tested can be directly involved in inhibition of anti-oxidant enzymes [181]. Overproduction of ROS is accompanied by inactivation of detoxification systems, consumptions of antioxidants and insufficient replenishment of antioxidants in cells/tissues [179]. However, the opposite trend with increased CAT activity is response to oxidizing agent exposure, was observed [211], and VitE doses were reported to decrease levels of erythrocyte antioxidant enzymes in human and animal studies [230]. Moreover, increased expression and activity of antioxidant enzymes was concluded to be a result of compensatory defence mechanisms against oxidative stress [225]. Nevertheless, the majority of literature data show that the presence of anti-oxidants (β-C, Vit E, Vit C) increased SOD, CAT and GPx activity in stressed cells, proving the ROS-scavenging properties of tested molecules.

### 7.3. Measurement of the Oxidative Stress in Cells and Tissues

The induction of oxidative stress in cells/tissues is typically measured by monitoring the cellular level of ROS (direct techniques) or the level of specific markers of oxidative stress, such as lipid peroxidation, protein modification and DNA oxidation products (indirect techniques). These techniques are used (or can be used) to evaluate the efficacy of various anti-oxidant molecules, such as β-carotene, vitamin E and ascorbic acid, on cells/tissues exposed to oxidative stress.

#### 7.3.1. Measurement of ROS Level: ESR Spectroscopy

Electron spin resonance (ESR) spectroscopy is a technique based on the absorption of microwave radiation by unpaired electron-possessing molecules (radicals) situated in the applied static magnetic field [241]. ESR spectroscopy is able to monitor the real-time generation of short-lived ROS radicals (O_2_^•−^, •OH) when coupled with spin traps. Spin-trapping reagents (nitrone and nitroso compounds) react with ROS radicals to yield long-lived radicals called spin-adducts [242]. 5,5-dimethyl-1-pyrroline N-oxide (DMPO), a commonly used nitrone, reacts with O_2_^•−^ or •OH to form DMPO-OOH or DMPO-OH radicals, respectively, while DMPO-OOH further decomposes to DMPO-OH [243]. Free radicals can be determined by analysing the distinct ESR spectrum of a spin adduct [242]. Apart from spin traps, ESR exploits spin probes (such as cyclic hydroxylamines) that undergo one-electron oxidation to form nitroxide radical detected by ESR, with higher sensitivity than spin traps [244]. ESR technique was used to measure ROS generation in oxidative stress-exposed human skin fibroblasts (CCD 1112Sk). 1-hydroxy-3-methoxy-carbonyl-2,2,5,5-tetramethyl pyrrolidine (CMH), as a spin probe, was used to react with ROS and to yield a stable nitroxide CM-radical, while ROS formation was measured according to the rate of nitroxide accumulation [197].

#### 7.3.2. Measurement of ROS Level: Fluorescent Method (DCFH-DA Probe Assay)

The intracellular level of ROS can be measured by means of the DCFH-DA probe assay. 2′,7′-dichlorodihydrofluorescein diacetate (DCFH-DA) (Figure S1) is a non-fluorescent compound that diffuses through the cellular membrane and undergoes the cleavage at the two ester bonds via intracellular esterase to 2′,7′-dichlorodihydrofluorescein (DCFH) which accumulates in cells. The non-fluorescent DCFH undergoes oxidation by ROS, such as H_2_O_2_, hydroxyl radicals and peroxyl radicals, to fluorescent 2′,7′-dichlorofluorescein (DCF). The presence of DCF can be detected fluorometrically with λ_excitation_ = 485 nm and λ_emission_ = 525 nm [245,246]. ROS detection with DCFH-DA faces possible limitations such as insufficient accessibility of esterases sequestered within cells, oxidation reaction with other cellular components, a lack of ability to react with O_2_^•−^ or the leakage of DCF out of the cells [245,246]. DCFH-DA probe assay is commonly used to evaluate the anti-oxidant effect of vitamins towards OS-induced cells (Table 2, Table 3 and Table 4).

#### 7.3.3. Measurement of ROS Level: Other Methods

O_2_^•−^ and H_2_O_2_ can be measured by means of chemiluminescence method, where light is emitted as a result of chemical reactions. H_2_O_2_ reacts in the presence of catalyst with luminol (3-aminophthalhydrazide) to produce the excited 3-aminophthalate anion, that emits light when relaxed to the ground state [247]. O_2_^•^^−^ reaction with lucigenin (N,N′-dimethyl-9,9′-biacridinium dinitrate, Luc^2+^) includes one-electron reduction of Luc^2+^ to lucigenin cation radical (Luc^•+^) that reacts with O_2_^•−^ to form lucigenin dioxetane (LucO_2_) which decomposes to N-methylacridone (NMA) with light emission [248].

O_2_^•−^ can be detected via oxidation of adrenaline to adrenochrome, that can be determined spectrophotometrically at 480 nm [249].

#### 7.3.4. Measurement of Lipid Peroxidation Products

Lipid peroxidation (LPO), the structural degradation of lipids occurring as a consequence of oxidative damage, is a widely used marker for OS presence. LPO is determined by measuring the level of malondialdehyde (MDA), 4-hydroxynonenal (4-HNE) or F_2_-isoprostanes (F_2_-IsoPs).

##### Measurement of Lipid Peroxidation: MDA, 4-HNE

MDA, a three-carbon dialdehyde, and 4-HNE, an unsaturated aldehyde, are products of oxidative degradation of polyunsaturated fatty acid (PUFAs), where double bonds in PUFAs structures are the target for ROS attack to form unstable fatty acid peroxides. 

The MDA level is measured spectrophotometrically, and the principle of method is the reaction (Figure S2) between MDA and 4,6-dihydroxypyrimidine-2-thiol (thiobarbituric acid; TBA) to form MDA-TBA2 adduct, that absorbs strongly at λ = 532 nm. Alternatively, MDA-TBA2 adduct can be measured spectrofluorometrically (λ_excitation_ = 515 nm/λ_emission_ = 553 nm) [250]. MDA assay is the most common method used to monitor OS level and evaluate the anti-oxidant effect of β-carotene, vitamin E and ascorbic acid on cells/tissues subjected to oxidative stress (Table 2, Table 3, Table 4 and Table 5). However, MDA determination based on TBA assay, lacks selectivity due to dependence on reaction conditions (pH, temperature) as well as reaction of TBA with other components of biological samples such as sugars, amino acids, bilirubin, albumin and other aldehydes, known as thiobarbituric acid reactive substances (TBARS) [250,251].

Improvement in MDA detection is achieved by using techniques of Chromatography and Mass Spectrometry, where molecules are analysed based on chromatogram retention time and mass to charge (m/z) ratio spectrum. MDA, after reaction with TBA, was reported to be analysed by HPLC-UV-Vis [252] or HPLC coupled with fluorescence detection [253]. MDA also undergoes derivatization with 2,4-dinitrophenylhydrazine (DNPH) to yield hydrazone and pyrazole (Figure S3) derivatives (MDA-DNPH) that can be analysed by HPLC-UV [254] or LC-MS [255]. Analysis of MDA by GC-MS requires derivatization of MDA with pentafluorobenzyl bromide (PFBB) or pentafluorobenzyl hydroxylamine (PFBHA) to form MDA-PFB2 (Figure S4) or MDA-PFB2-oxime (Figure S5) derivatives, respectively [256]. 

Low density lipoproteins (LDL) modified by MDA (LDL-MDA), markers of atherosclerosis development, can be determined by the enzyme-linked immunosorbent assay (ELISA). The principle of this assay is the reaction of the specific monoclonal antibody 3B2 with MDA-LDL in plasma, the capture of the complex by anti-IgM antibody coated to an ELISA plate, the binding of polyclonal anti-(MDA-LDL) antibody, the binding of anti-rabbit IgG (secondary antibody) conjugated with horseradish peroxidase (HRP) and the reaction of HRP with o-phenylenediamine (OPD) to yield a product detected spectrophotometrically [257].

4-HNE can be analysed by means of GC-MS, upon derivatization (Figure S6) with PFBHA to yield 4-HNE-PFB-oxime product, that subsequently undergoes silylation by *N,O*-bis(trimethylsilyl)trifluoroacetamide (BSTFA) in trimethylchlorosilane (TMCS) to form 4-HNE-PFB-oxime-TMS derivative [197,256].

4-HNE protein adducts can be analysed by ELISA, involving the anti-HNE antibody and HRP-labelled secondary antibody (indirect ELISA) or HRP-labelled anti-HNE antibody (Sandwich ELISA), and tetramethylbenzidine (TMB) as a HRP-specific substrate [258]. 

##### Measurement of Lipid Peroxidation: F_2_-Isoprostanes (F_2_-IsoPs)

Isoprostanes (IsoPs) are prostaglandin (PG)-like compounds formed by non-enzymatic free-radical mediated peroxidation of arachidonic acid. The 11-, 9-, 12- or 8-peroxidation of arachidonic acid leads to formations of F_2_-isoprostanes classified as 15-series, 5-series, 8-series or 12-series F_2_-IsoPs, respectively [251]. 

F_2_-IsoPs are considered as reliable biomarkers of oxidative stress and can be measured by liquid chromatography-mass spectrometry (LC-MS), where F_2_-IsoPs molecules are detected based on chromatogram retention time and mass to charge (m/z) ratio spectrum [259]. F_2_-IsoPs can be also measured by gas chromatography-mass spectrometry (GC-MS), and F_2_-isoprostanes undergo reaction (Figure S7) with pentafluorobenzyl bromide (PFBB) to form pentafluorobenzyl (PFB) esters, and react with N,O-bis(trimethylsilyl)trifluoroacetamide (BSTFA) to form trimethylsilyl (TMS) ether derivatives of F_2_-IsoPs PFB esters, prior to GC-MS analysis [260].

F_2_-IsoPs can be also determined by means of commercially available enzyme-linked immunosorbent assay (ELISA) kit [253], or by radioimmunoassay technique [229,261,262].

Detection of isoprostanes were applied during in vitro (Table 4) and populational (Table 6) OS studies.

##### Measurement of Lipid Peroxidation: Lipid Hydroperoxides

Lipid hydroperoxides (LOOHs) are the initial products formed as a result of the peroxidation of unsaturated fatty acids. Hydroperoxides can be measured via ferrous oxidation in xylenol orange (FOX) assay, which is based on oxidation of Fe^2+^ to Fe^3+^ by hydroperoxides under acidic conditions and complexation of Fe^3+^ by xylenol orange (XO) to form a blue-purple complex with an absorbance maximum at 550–600 nm. For plasma samples, FOX assay is authenticated by triphenylphosphine (TPP) which selectively reduces the hydroperoxides into their corresponding alcohols, whereby providing a control and eliminating the interference from plasma components (Fe^3+^) [263,264].

#### 7.3.5. Measurement of Protein Oxidation: Protein Carbonyls

Protein-bound carbonyls are the most commonly used biomarker for protein oxidation. Protein carbonyls originate from oxidative cleavage of the protein structure, direct oxidation of amino acids (lysine, arginine, histidine, proline, glutamic acid, threonine), or introducing carbonyl groups via reaction of lipid-oxidation derived-aldehydes with cysteine (Cys), histidine (His), arginine (Arg) and lysine (Lys) residues. 

The principle of protein carbonyl measurement is the use of 2,4-dinitrophenylhydrazine (DNPH) that reacts with protein carbonyl groups producing a protein carbonyl-DNP hydrazone (Figure S8), which can be detected spectrophotometrically at λ = 360–390 nm [265,266] or by HPLC-UV [252]. DNPH-derivatized protein carbonyls can be also detected by immunoblotting technique, with antibodies specific to the DNP moiety of the proteins [70,173]. Protein carbonyls were determined during in vitro (Table 3 and Table 4) and in vivo (Table 5) OS studies.

#### 7.3.6. Measurement of DNA Oxidation: 8-OHdG

Oxidation of DNA is commonly measured based on biomarkers such as 8-hydroxydeoxyguanosine (8-OHdG), the major form of oxidative deoxyribonucleic acid (DNA) damage [267]. 

The standard technique to detect 8-OHdG is the enzyme-linked immunosorbent assay (ELISA). The principle of this assay (Figure S9) comprises binding of antibody to 8-OHdG, subsequent binding of horse radish peroxidase (HRP)-conjugated secondary antibody to the anti(8-OHdG) antibody, conversion of tetramethylbenzidine (TMB) via HRP in the presence of H_2_O_2_ to a product and termination of reaction with the use of phosphoric acid. The product formed as a result of TMB conversion can be quantified spectrophotometrically at λ = 450 nm [268]. Such a technique was used to monitor oxidative stress in HEK293 cells exposed to H_2_O_2_ and ascorbic acid [193].

8-OHdG can be also detected by means of HPLC with electrochemical detection (HPLC-ECD) [269], or by LS-MS techniques [270]. A technique of LC-MS was used to detect and quantify 8-OHdG in OS-exposed human skin fibroblasts (CCD 1112Sk) [197].

#### 7.3.7. Analysis of Oxidative Stress and Vitamin Effect by Raman Spectroscopy and Imaging

**Figure 7:**
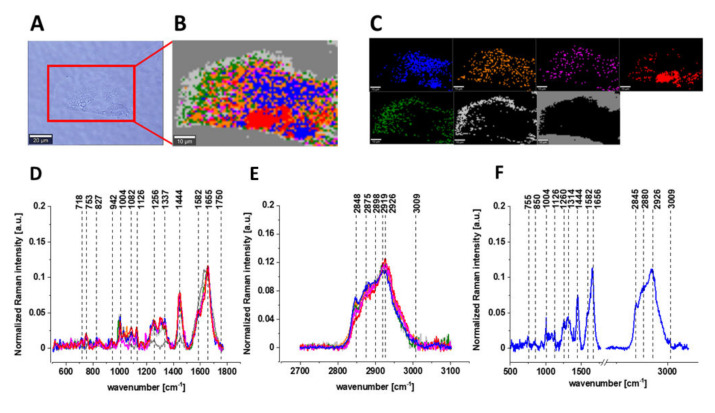
The microscopy image of exemplary CCD-18Co cell (**A**), Raman image constructed based on Cluster Analysis (CA) method (**B**), Raman images of all clusters identified by CA assigned to: nucleus (red), mitochondria (magenta), lipid-rich regions (blue, orange), membrane (light grey), cytoplasm (green), and cell environment (dark grey) (**C**), average Raman spectra typical for all clusters identified by CA in a 500–1800 cm^−^^1^ (**D**) and a 2700–3100 cm^−^^1^ (**E**) wavenumber region, average Raman spectrum for the whole cell within 500–3100 cm^−1^ (**F**); cells measured in PBS, excitation wavelength: 532 nm. Reprinted with permission from [277].

The effect of oxidative stress and vitamin supplementation in cells can be also evaluated by means of Raman spectroscopy and imaging. Raman spectroscopy, is a technique exploiting the ability of a molecule to vibrate and to inelastically scatter (Raman effect) absorbed light [271]. A source of light to be absorbed by molecules are lasers emitting monochromatic light from ultraviolet (355 nm), green (532 nm), red (633 nm) or near-infrared (785 nm, 1064 nm) regions of electromagnetic radiation [271]. The principle of Raman spectroscopy technique is to analyse the Raman scattered light for chemical and structural characterization of investigated samples [201]. It is based on the fact that chemical bonds (C-H, C-C, C=C, C-N, C=O, N-H, -O-P-O-, etc.) vibrate in their specific modes and, when interacting with light, produce specific spectral bands with different Raman shift. Raman spectroscopy enables structural description of single molecules and polymers as well as higher macroscopic structures, based on spectral profiles in fingerprint (500–1800 cm^−^^1^) and high wavenumber (2700–3100 cm^−1^) region [272]. Raman spectroscopy can be coupled with optical confocal microscopy, enabling to conduct high-resolution chemical imaging. Raman spectroscopy and imaging have been used for structural investigation of different types of cells, including brain [273], breast [274] and colon [275] cells. Raman spectroscopy and imaging were used by our team for structural characterization of human fibroblast colon (CCD-18Co) cells (Figure 7). Raman spectra contain bands assigned to specific chemical structures [276], based on vibrational features of molecules within an analysed cell. The presence of nucleic acids is detected due to Raman bands at 716–723 cm^−1^ (nucleotides, DNA), 781–787 cm^−^^1^ (nucleotides, DNA, RNA), 1070–1093 cm^−1^ (PO_2_^−^, PO_4_^3−^). Protein presence can be concluded based on Raman bands at 748–757 cm^−1^ (tryptophan), 852–858 cm^−1^ (proline, hydroxyproline, tyrosine), 992–1010 cm^−1^ (phenylalanine), 1263–1272 cm^−1^ (Amide III) and 1658–1664 cm^−1^ (Amide I), and 2926 cm^−1^ (CH_3_). The presence of lipids is determined according to Raman bands at 1127–1133 cm^−1^ (acyl backbone, fatty acids), 1299–1305 cm^−1^ (acyl chains, fatty acids), 1440–1444 cm^−1^ (CH_2_, CH_3_, fatty acids, cholesterol), 2854 cm^−1^ (CH_2_) and 3009 cm^−1^ (=CH). Particular signals in spectra are ascribed to nucleic acids, proteins and/or lipids, thereby providing structural characterization of CCD-18Co cells. Furthermore, Raman images of individual cells can be constructed with the use of Cluster Analysis (CA) method, where Raman images of all clusters identified by CA are assigned to: nucleus, mitochondria, lipid-rich regions, membrane, cytoplasm, and cell surroundings, within spectral wavenumber (500–3100 cm^−1^) region.

**Figure 8:**
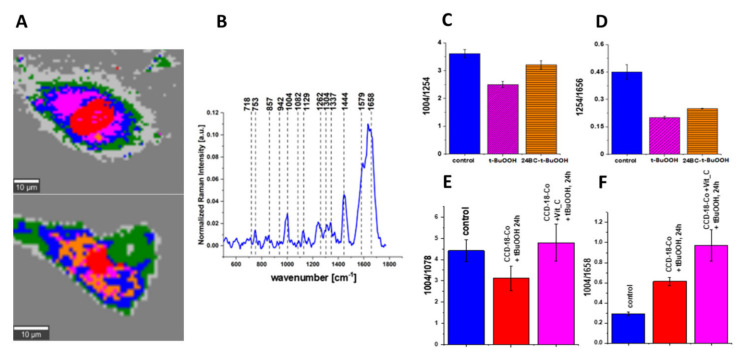
Raman spectroscopy analysis of cells exposed to t-BuOOH, β-carotene and/or Vit C; Raman image of CCD-18Co exemplary cells (**A**), average Raman spectra of exemplary CCD-18Co cell (**B**), Raman I_1004/1254_ (**C**) and Raman I_1254/1656_ (**D**) graph values with bars: control, t-BuOOH, t-BuOOH + β-C, Raman I_1004/1078_ (**E**) and Raman I_1004/1658_ (**F**) graph values with bars: control, t-BuOOH, t-BuOOH + Vit C. Reprinted and adapted with permission from [277,278].

Raman spectroscopy and imaging can be further used to monitor structural and metabolic alterations in cells exposed to oxidative stress (t-BuOOH) and/or treatment with antioxidants (β-carotene, ascorbic acid) (Figure 8). Raman spectroscopy measurement of such investigated cells provide spectral pattern with specific intensities of different bans in the spectrum. A ratio of intensities from different bands within spectrum can provide further information regarding molecular characterization of cells. Different ratios, for selected Raman band intensities corresponding to 1004/1254 (phenylalanine/amide III proteins), 1254/1656 (amide III/amide I proteins), 1004/1078 (phenylalanine/nucleic acids and phospholipids) and 1004/1658 (phenylalanine/amide I proteins), were determined by our group to monitor metabolic alterations in cells. In one study, CCD-18Co cells were subjected to t-BuOOH as OS inducer and β-carotene as an antioxidant [278]. Results showed that Raman I_1004/1254_ and I_1254/1656_ values in CCD-18Co cells changed due to exposure to t-BuOOH or co-treatment with t-BuOOH and β-C, if compared to control CCD-18Co cells. In another study, CCD-18Co cells were exposed to t-BuOOH for oxidative stress induction and ascorbic acid to provide antioxidant protection [277]. Results showed that Raman I_1004/1078_ and I_1004/1658_ values in CCD-18Co cells altered due to exposure to t-BuOOH or co-treatment with t-BuOOH and AscA, if compared to control CCD-18Co cells [277]. Therefore, intensities of different bands within average spectra compared as ratios can provide structural and molecular characterization of cells subjected to oxidative/anti-oxidative treatment.

#### 7.3.8. Measurement of the Oxidative Stress: A Summary

OS increase during in vitro, in vivo and populational studies can be characterized by elevated level of direct and indirect specific (bio)markers, such as intracellular ROS level, fatty acids (lipids) oxidation products (MDA, 4-HNE, F_2_-isoprostanes), protein oxidation products (protein carbonyls) and DNA oxidation products (8-hydroxydeoxyguanosine). The measurements of biomarkers usually depend on the use of spectrophotometric, fluorometric, chromatographic techniques also coupled with mass spectrometry (LC-MS, GC-MS), as well as ELISA assay. However, the use of these techniques involves a series of cumbersome steps including cell disruption and extract preparation, its purification and separation into specific fractions and derivatization of target molecules with specific compounds prior to analysis. The alternative and/or support to these techniques could be Raman spectroscopy and imaging that enable the whole-cell characterization of different chemical structures (proteins, lipids, nucleic acids), without the necessity of cell disruption, and further fractionization and derivatization. Raman spectroscopy measurement can provide the monitoring of the alteration of cellular chemical profile, based on Raman spectra analysis, when cells are exposed to stress inducer and/or antioxidant treatment. Indeed, results published by our group show that chemical changes in colon CCD-18Co cells exposed to stress conditions and treated with anti-oxidant (β-carotene, ascorbate) can be monitored by Raman spectroscopy and imaging. The use of this technique can be further extended for characterization of other cells and studying the effect of new anti-oxidants.

## 8. Conclusions

In this review, antioxidant properties of β-carotene, tocopherols and ascorbic acid are presented based on in vitro, in vivo and populational/clinical studies. Literature data gathered suggests that β-carotene, tocopherols and ascorbic acid possess the potential to mitigate oxidative stress in various biological systems. However, interpretation of gathered results faces possible limitations due to different cells, animal species and OS-inducing agents used, discrepancies between in vitro and in vivo studies, as well as insufficient number of participants and various origins of oxidative stress in populational/clinical studies. Therefore, further studies are required to determine the effect of β-carotene, tocopherols or ascorbic acid on oxidative stress level and the efficacy of tested antioxidants in different biological systems (in vitro, in vivo animals, populational/clinical). Oxidative stress (bio)markers are measured by a range of different techniques (spectrophotometric, fluorometric, chromatographic, immuno-enzymatic). Apart from commonly used analytical methods, Raman spectroscopy and imaging can be a useful technique to study the effect of oxidative stress and anti-oxidant molecules in cell studies.

## Figures and Tables

**Table 1:** Exemplary structures of oxidation products of lipids, proteins and nucleic acids.

Exemplary Structures of Oxidation Products		
Name	Structure Description	Mechanism of Formation
Malondialdehyde (MDA)	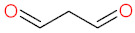	PUFA peroxyl radical undergoes intramolecular cyclization to endoperoxide with further breakdown to MDA
4-hydroxy-2-nonenal (4-HNE; HNE)	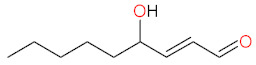	Peroxidation of n-6 PUFAs and the generation of α,β unsaturated aldehydes
Dityrosine	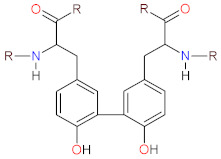	Generation of a tyrosyl radical, radical isomerization, diradical reaction, and enolization
HNE-Lys adduct (protein carbonyl product)	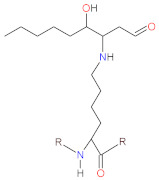	Michael addition of the HNE double bond to NH_2_-group of lysine (Lys)
HNE-Cys adduct (protein carbonyl product)	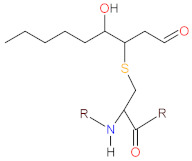	Michael addition of the HNE double bond to SH-group of cysteine (Cys)
HNE-His adduct (protein carbonyl product)	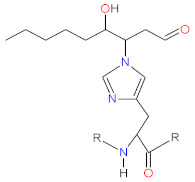	Michael addition of the HNE double bond to NH in an imidazole of histidine (His)
8-oxo-2′-deoxyguanosine nucleotide	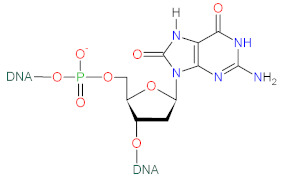	Reaction between C-8 of guanine (G) and hydroxyl radical (•OH)
8-oxo-2′-deoxyadenosine nucleotide	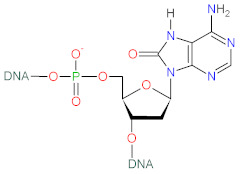	Reaction between C-8 of adenine (A) and hydroxyl radical (•OH)
5,6-dihydroxy-5,6-dihydrothymidine nucleotide	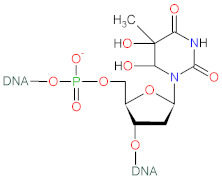	Reaction of hydroxyl radical (•OH) with C-5 and C-6 of thymine (T)
5,6-dihydroxy-5,6-dihydrocytidine nucleotide	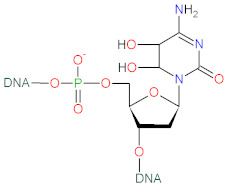	Reaction of hydroxyl radical (•OH) with C-5 and C-6 of cytosine (C)

**Table 2:** Antioxidant activity of β-carotene towards oxidative stress (OS) induced in animal and human cells due to exposure to various stress agents.

Antioxidant Effect of β-Carotene Towards OS-Induced Cells				
Cell Type	Oxidative Stress Inducer	Exposure Mode	Intracellular Effect	Ref.
Rat pheochromocytoma (PC12) cells (undifferentiated)	H_2_O_2_	Incubation: 12 h without β-C and then 12 h with 40 µM H_2_O_2_	↑*^1^* ROS	[153]
		Incubation: 12 h with 0.5–10 µM β-C and then 12 h with 40 µM H_2_O_2_	↓*^2^* ROS	
Rat hepatocytes	Glycocheno-deoxycholic acid (GCDC)	Incubation: 0.5 h without β-C and then 4 h with 100 µM GCDC	↑*^1^* ROS	[183]
		Incubation: 0.5 h with 50 µM β-C and then 4 h with 100 µM GCDC	↓*^2^* ROS	
Oocytes	Rosup	Incubation: 4 h with 33.3 µg/mL Rosup	↑*^1^* ROS	[184]
		Incubation: 4 h with 10 µM β-C and 33.3 µg/mL Rosup	↓*^2^* ROS	
Human erythrocytes	H_2_O_2_	Incubation: 0.5 h with 20 µM H_2_O_2_	↑*^1^* lipid peroxidation, ↓*^1^* SOD, ↓*^1^* CAT	[185]
		Incubation: 0.5 h with 3 µM β-C and 20 µM H_2_O_2_	↓*^2^* lipid peroxidation, ↑*^2^* SOD, ↑*^2^* CAT	
Mice erythrocytes	H_2_O_2_	Incubation: 2 mM H_2_O_2_	↑*^1^* MDA	[186]
		Incubation: 10 µg/mL β-C and 2 mM H_2_O_2_	↓*^2^* MDA	
Cardiomyocyte cells (H9c2)	AGEs	Incubation: 24 h with 200 µg/mL AGEs	↑*^1^* ROS, ↑*^1^* MDA, ↓*^1^* GPx, ↓*^1^* SOD	[187]
		Incubation: 24 h with 200 µg/mL AGEs + 24 h with 40 µM β-C	↓*^2^* ROS, ↓*^2^* MDA, ↑*^2^* GPx, ↑*^2^* SOD	
Normal and tumor thymocytes	t-BuOOH	Incubation: 2 h with 0.5 mM t-BuOOH	↑*^1^* MDA	[188]
		Incubation: 0.5 h with β-C (2.8 nmol/mg dry wt) and 2 h with 0.5 mM t-BuOOH	↓*^2^* MDA	

*^1^*—if compared to control without OS inducer used; *^2^*—if compared to profile with OS inducer used; Measurement methods of ROS/oxidative stress markers: ROS: DCFA-DA; Lipid peroxidation: TBARS; MDA:TBA.

**Table 3:** Antioxidant activity of tocopherols/vitamin E towards oxidative stress (OS) induced in animal and human cells due to exposure to various stress agents.

Antioxidant Effect of Vitamin E towards OS-Induced Cells				
Cell Type	Oxidative Stress Inducer	Exposure Mode	Exposure Effect	Ref.
Human keratinocyte cells	UVA	Irradiation (UVA, 8 J/cm^2^) + incubation for 24 h	↑*^1^* ROS, ↑*^1^* MDA	[189]
		Incubation for 24 h with α-T (2.9–14.7 IU/mL), irradiation (UVA, 8 J/cm^2^) + incubation for 24 h	↓*^2^* ROS, ↓*^2^* MDA	
Human neuroblastoma (SH-SY5Y) cells	AGEs	Incubation for 24 h without α-T, then incubation with 1.5 mg/mL AGEs for 72 h	↑*^1^* lipid peroxidation, ↑*^1^* protein carbonyls	[173]
		Incubation for 24 h with α-T (200 µM), then incubation with 1.5 mg/mL AGEs for 72 h	↓*^2^* lipid peroxidation, ↓*^2^* protein carbonyls	
Human umbilical vein endothelial cells	Hcy	Incubation with Hcy (1 mM)	↑*^1^* ROS, ↑*^1^* MDA	[172]
		Incubation with Hcy (1 mM) and VitE (50 µM)	↓*^2^* ROS, ↓*^2^* MDA	
Human erythrocytes	Dichlorvos (DDVP)	Incubation with DDVP (10 µM)	↑*^1^* MDA, ↓*^1^* SOD, ↓*^1^* CAT, ↓*^1^* GPx	[190]
		Incubation with DDVP (10 µM) and Vit E (30 µM)	↓*^2^* MDA, ↑*^2^* SOD, ↑*^2^* CAT, ↑*^2^* GPx	
Human colorectal adenocarcinoma cell line (Caco-2)	H_2_O_2_	Incubation for 24 h, incubation for 48 h with H_2_O_2_ (250 µM)	↑*^1^* MDA	[191]
		Incubation for 24 h, incubation for 48 h with H_2_O_2_ (250 µM) and VitE (10 µM)	↓*^2^* MDA	
Rat hepatocytes	Glycocheno-deoxycholic acid	Incubation: 0.5 h without α-T and then 4 h with 100 µM GCDC	↑*^1^* ROS	[183]
		Incubation: 0.5 h with 100 µM α-T and then 4 h with 100 µM GCDC	↓*^2^* ROS	
Rat renal tubular epithelial cells (NRK-52E)	Aristolochic acid (AA)	Incubation: AA (10 µM)	↑*^1^* H_2_O_2_	[192]
		Incubation: AA (10 µM) + α-T (5–100 µM)	↓*^2^* H_2_O_2_	

*^1^*—if compared to control without OS inducer used; *^2^*—if compared to profile with OS inducer used; Measurement methods of ROS/oxidative stress markers: ROS: DCFA-DA; Lipid peroxidation: TBARS; MDA: TBA; Protein carbonyls: Immunoblotting; H_2_O_2_: Chemiluminescence.

**Table 4:** Antioxidant activity of ascorbic acid towards oxidative stress (OS) induced in animal and human cells due to exposure to various stress agents.

Antioxidant Effect of Ascorbic Acid towards OS-Induced Cells				
Cell Type	Oxidative Stress Inducer	Exposure Mode	Exposure Effect	Ref.
Human embryonic kidney (HEK293) cells	H_2_O_2_	Incubation: 4 h with H_2_O_2_ (400 µM)	↑*^1^* ROS, ↑*^1^* MDA, ↓*^1^* CAT, ↓*^1^* SOD, ↓*^1^* GPx, ↑*^1^* protein carbonyl, ↑*^1^* 8-OHdG	[193]
		Incubation: 24 h with Vit C (1–20 µM), and then 4 h with H_2_O_2_ (400 µM)	↓*^2^* ROS, ↓*^2^* MDA, ↑*^2^* CAT, ↑*^2^* SOD, ↑*^2^* GPx, ↓*^2^* protein carbonyl, ↓*^2^* 8-OHdG	
Human lens epithelial cells (LEC)	H_2_O_2_	Incubation: 24 h without VitC, and 0.5 h with H_2_O_2_ (0.2 mM)	↑*^1^* ROS	[194]
		Incubation: 24 h with VitC (1 mM), and 0.5 h with H_2_O_2_ (0.2 mM)	↓*^2^* ROS	
Human retinal pigment epithelial (ARPE-19) cells	H_2_O_2_ or UVB	Incubation: UVB irradiation (20–100 mJ/cm^2^) Incubation: 24 h with H_2_O_2_ (0.2 mM)	↑*^1^* ROS	[195]
		Incubation: 6 h with VitC (500 µM), then UVB irradiation (100 mJ/cm^2^) Incubation: 6 h with VitC (20 µM), then 24 h with H_2_O_2_ (0.2 mM)	↓*^2^* ROS	
Human erythrocytes	Dichlorvos	Incubation with DDVP (10 µM)	↑*^1^* MDA, ↓*^1^* SOD, ↓*^1^* CAT, ↓*^1^* GPx	[190]
		Incubation with DDVP (10 µM) and Vit C (10 µM)	↓*^2^* MDA, ↑*^2^* SOD, ↑*^2^* CAT, ↑*^2^* GPx	
Human hepatoma (HepG2) cells	Ethanol, sodium selenite or t-BuOOH	Incubation with ethanol (10–500 µM) or sodium selenite (1–10 µM) or t-BuOOH (20–200 µM) for 24 h	↑*^1^* MDA ↓*^1^* SOD, ↓*^1^* CAT, ↓*^1^* GSH	[168]
		Cotreatment with Vit C (25–100 µM) and one of OS inducer for 24 h	↓*^2^* MDA, ↑*^2^* SOD, ↑*^2^* CAT, ↑*^2^* GSH	
Human skin fibroblasts (CCD 1112Sk)	UVA, UVB or H_2_O_2_	For irradiation treatment: 20 J/cm^2^ (UVA) or 200 mJ/cm^2^ (UVB) + 24 h incubation; For H_2_O_2_ treatment: incubation with 200 µM H_2_O_2_ for 24 h	↑*^1^* ROS, ↑*^1^* MDA, ↑*^1^* 4-HNE, ↑*^1^* Carbonyl groups (for all stress inducers); ↑*^1^* Isoprostanes (for UVA, H_2_O_2_)	[197]
		Stress induction treatment + 24 h incubation with 100 µM ascorbic acid	↓*^2^* ROS, ↓*^2^* MDA, ↓*^2^* 4-HNE, ↓*^2^* Carbonyl groups (for all stress inducers); ↓*^2^* Isoprostanes (for UVA, H_2_O_2_)	
Rat renal tubular epithelial cells (NRK-52E)	Aristolochic acid (AA)	Incubation: AA (10 µM)	↑*^1^* H_2_O_2_	[196]
		Incubation: AA (10 µM) + Vit C (5 µM)	↓*^2^* H_2_O_2_	

*^1^*—if compared to control without OS inducer used; *^2^*—if compared to profile with OS inducer used; Measurement methods of ROS/oxidative stress markers: ROS: DCFA-DA, ESR [197]; MDA:TBA, GC-MS [197]; 4-HNE: GC-MS [197]; Isoprostanes: LC-MS [197]; Protein carbonyls: DNPH; 8-OHdG: ELISA [193], LC-MS [197]; H_2_O_2_: Chemiluminescence.

**Table 5:** Antioxidant activity of β-carotene, vitamin E and/or vitamin C based on animal in vivo studies with different oxidative stress inducers applied.

Antioxidant Effect of β-Carotene, Vitamin E and/or Vitamin C In Vivo				
Species/Tissue	Oxidative Stress Inducer	Exposure Mode	Exposure Effect	Ref.
Wistar rats n = 24 (total)/Liver tissue	Methotrexate (MTX)	Single MTX dose (20 mg/kg) on day 21 of experiment (24 days)	↑*^1^* MDA, ↓*^1^* SOD, ↓*^1^* CAT, ↓*^1^* GPx	[201]
		β-C dose (10 mg/kg/day) for 24 days + MTX dose (20 mg/kg) on day 21	↓*^2^* MDA, ↑*^2^* SOD, ↑*^2^* CAT, ↑*^2^* GPx	
Wistar rats n = 30 (total)/Blood	High-fat diet (HFD) (Mixing of cow fat (60%) with normal rat chow (40%))	HFD for 14 weeks + 24 h starving	↑*^1^* MDA	[200]
		β-C administration (300 mg/kg body weight) for 2 weeks before or after 12-week HFD + 24 h starving	↓*^2^* MDA	
Male Sprague–Dawley rats n = 299 (total)/Spinal Cord tissue	Spinal Cord Injury (SCI)	SCI surgery + 72 h	↑*^1^* MDA, ↓*^1^* SOD	[202]
		SCI surgery + β-C (20–80 mg/kg) administered intraperitoneally once immediately after the surgery + 72 h	↓*^2^* MDA, ↑*^2^* SOD	
Male C57BL/6 mice n = 108 (total)/Brain tissue	Traumatic Brain Injury (TBI)	TBI surgery + 7 days	↑*^1^* MDA, ↓*^1^* SOD	[203]
		TBI surgery + β-C (30 mg/kg) administered 3 h after the surgery and then every day during 7 days	↓*^2^* MDA, ↑*^2^* SOD	
*Drosophila melanogaster* larvae	Gamma irradiation	Exposure to 10 Gy γ-irradiation	↑*^1^* Lipid peroxidation	[204]
		Larvae feeding on β-C before exposure to 10 Gy γ-irradiation	↓*^2^* Lipid peroxidation	
Wistar male rats n = 60 (total)/Serum	Cd^2+^ (CdCl_2_·H_2_O)	Single intraperitoneal injection of Cd^2+^ ions (2 mg Kg^−1^)	↑*^1^* Lipoperoxide, ↓*^1^* SOD	[210]
		Single intraperitoneal injection of Cd^2+^ ions (2 mg/Kg) + administration of drink aqueous solutions of α-T (40 mg/L) for 15 days	↓*^2^* Lipoperoxide, ↑*^2^* SOD	
Male Sprague–Dawley rats n = 70 (total)/Heart tissue	Doxorubicin (DOX)	Intraperitoneal injection (4 mg DOX/ kg body weight) three times per week for 2 weeks	↑*^1^* MDA, ↑*^1^* CAT	[211]
		Intra-gastric administration (100 mg VitE/kg body weight), two times per week for 3 weeks, started 1 week before DOX injection	↓*^2^* MDA, ↓*^2^* CAT	
Female white mice n = 160 (total)/Blood	Cyfluthrin	Oral administration of cyfluthrin (a single dose of 100 mg/kg/body weight)	↑*^1^* MDA, ↓*^1^* CAT	[181]
		Oral administration of cyfluthrin (a dose of 100 mg/kg/body weight) followed by intramuscular injection of VitE (a dose of 100 mg/kg/body weight, for 7 days)	↓*^2^* MDA, ↑*^2^* CAT	
C57BL/6 mice/Infarcted tissue	Ischemia/Reperfusion (I/R) injury	I/R injury + 3 days	↑*^1^* Oxidized lipids	[212]
		Intraperitoneal injection of α-TOH (2.5 mg/kg BW) 2 h before surgery, immediately after reperfusion and twice per day for 3 days	↓*^2^* Oxidized lipids	
BALB/c mice n = 40 (total)/Heart tissue	Heat stress (HS)	HS conditions (temperature: 40 °C; humidity: 60%) for 4 h per day during a 4-week period	↑*^1^* MDA, ↓*^1^* SOD	[213]
		Oral administration of VitE (500 mg/kg) 2 h before the initiation of HS	↓*^2^* MDA, ↑*^2^* SOD	
Sprague-Dawley male rats n = 30 (total)/Blood	High-fat diet	A 10-week feeding on high-fat diet	↑*^1^* Lipid peroxidation, ↓*^1^* SOD, ↓*^1^* GPx	[214]
		A 10-week feeding on high-fat diet supplemented with VitE (350 mg/kg diet)	↓*^2^* Lipid peroxidation, ↑*^2^* SOD, ↑*^2^* GPx	
Ross 308 male broilers (21-day old) n = 400 (total)/Blood, Breast muscle	High n-3 dietary PUFAs intake	Chickens fed with commercial starter diet (1–12 days), commercial grower diet (13–20 days), finisher diet enriched with 5% cold-pressed linseed oil and supplemented with VitE (200 IU/kg) (21–40 days)	↓*^3^* MDA	[215]
Wistar rats n = 28 (total)/Stomach, Colon, Kidney tissue	Sodium azide (NaN_3_)	Oral administration of NaN_3_ (20 mg/kg BW) for 9 days	↑*^1^* MDA, ↑*^1^* Protein Carbonyls	[222]
		Oral administration of NaN_3_ and VitC (200 mg/kg BW) for 9 days	↓*^2^* MDA, ↓*^2^* Protein Carbonyls	
Wistar male rats n = 46/Heart tissue	Doxorubicin (DOX)	Six intraperitoneal DOX injections (2.5 mg/kg body wt) over 3 weeks	↑*^1^* Superoxide anion, ↑*^1^* Lipid peroxidation, ↑*^1^* Protein Carbonyls	[223]
		Oral daily administration of VitC (50 mg/kg) started 1 week before the start of DOX administration and continued for 2 weeks after the last DOX injection	↓*^2^* Superoxide anion, ↓*^2^* Lipid peroxidation, ↓*^2^* Protein Carbonyls	
Sprague–Dawley male rats n = 18 (total)/Heart tissue	Quinalphos (QP)	Oral dose of QP (14 mg/kg), daily for 10 days	↑*^1^* MDA, ↓*^1^* CAT, ↓*^1^* GPx	[224]
		Oral administration of VitC (20 mg/kg) daily, 4 h after QP administration, for 10 days	↓*^2^* MDA, ↑*^2^* CAT, ↑*^2^* GPx	

*^1^*—if compared to control without OS applied; *^2^*—if compared to profile with OS applied; *^3^*—if compared to profile without anti-oxidant applied; Measurement methods of ROS/oxidative stress markers: Lipid peroxidation: TBARS; MDA: TBA; Protein carbonyls: DNPH, Oxidized lipids: LC-MS; Superoxide anion: Adrenaline assay.

**Table 6:** Antioxidant activity of β-carotene, vitamin E and/or vitamin C based on populational/clinical studies.

Antioxidant Effect of β-Carotene, Vitamin E and/or Vitamin C in Human Studies				
Patients/Participants	Origin of Oxidative Stress	Treatment Mode	Treatment Effect	Ref.
Male workers exposed to lead, n = 85 (total)/Blood	Pb	No administration of antioxidants for 12 weeks	-	[225]
		Oral administration of β-carotene (10 mg/day) for 12 weeks	↓*^1,2^* MDA, ↓*^1,2^* LHP	
Patients with NIDDM, n = 20 (total)/Blood	NIDDM	Supplementation with β-carotene (60 mg/day) for 3 weeks	↓*^C1^* MDA	[226]
Participants with polygenic hypercholesterolemia, n = 35/Blood	Enhanced oxidative stress	Supplementation with placebo for 16 weeks	-	[227]
		Supplementation with VitE (1600–3200 I.U.) for 16 weeks	↓*^2^* F_2_-isoprostane	
White volunteers, n = 8 (total)/Skin	UVR	Supplementation with α-tocopherol (400 IU/day) for 8 weeks, skin exposure to UVR (120 mJ/cm^2^), skin biopsy 6 h after UVR exposure	↓*^2^* MDA	[228]
Participants at age of 77, n = 704/Urine	n.d.	Dietary intake of ascorbic acid and β-carotene	↓*^I^* F_2_-isoprostane	[229]
Female participants with type 2 diabetes, n = 34 (total)/Blood	Type 2 diabetes	Oral administration of placebo (800 mg/day) for 6 weeks	-	[230]
		Oral administration of α-tocopherol (800 IU/day) for 6 weeks	↓*^1,2^* MDA	
Participants with DMT2, n = 5 or 8 (total)/Blood	DMT2	Supplementation with VitC (1000 mg/day) for 6 weeks	↓*^1^* MDA ↓*^1^* F_2_-isoprostane	[231]
Participants with DMT2 and administered with hypoglycemic drug, n = 80 (total)/Blood	DMT2	No supplementation of VitE within 3 months	↑*^1,C2^* MDA	[232]
		Supplementation of VitE (400 mg/day) for 3 months	↓*^1,2^* MDA	
Patients with late-stage knee osteoarthritis, n = 72 (total)/Blood, synovial fluid	Enhanced oxidative stress	Oral administration of placebo once daily for 2 months	-	[233]
		Oral administration of VitE (400 IU/day) for 2 months	↓*^2^* MDA	

*^1^*—if compared to the beginning of the study; *^2^*—if compared to profile without antioxidant used; *^C1^*—if compared to control: normoglycemic profile; *^C2^*—if compared to control: healthy subjects; *^I^*—if compared to lower intake of antioxidants; Measurement methods of oxidative stress biomarkers: MDA: TBA; LHP: FOX assay; F_2_-Isoprostanes: GC-MS [227], Radioimmunoassay [229], ELISA [231].

## Supplementary Materials

The following are available online at https://www.mdpi.com/article/10.3390/biom12081087/s1, Figure S1: The mechanism of DCFH-DA conversion to DCFH and DCF; Figure S2: The mechanism of reaction between MDA and TBA to form MDA-TBA2; Figure S3: The mechanism of reaction between MDA and DNPH to form MDA-DNPH; Figure S4: The mechanism of reaction between MDA and PFBB to form MDA-PFB2; Figure S5: The mechanism of reaction between MDA and PFBHA to form MDA-PFB2-oxime; Figure S6: The mechanism of 4-HNE-PFB-oxime-TMS formation and analysis. PFBHA: pentafluorobenzyl hydroxylamine; BSTFA: N,O-bis(trimethylsilyl)trifluoroacetamide; TMCS: trimethylchlorosilane; TMS: trimethylsilyl; Figure S7: The mechanism of F_2_-isoprostane (F_2_-IsoPs) formation and analysis. PFBB: pentafluorobenzyl bromide; BSTFA: N,O-bis(trimethylsilyl)trifluoroacetamide; TMS: trimethylsilyl; Figure S8: The mechanism of protein carbonyl reaction with dinitrophenylhydrazine (DNPH). Figure S9. The mechanism of deoxyguanosine (dG) oxidation into 8-hydroxydeoxyguanosine (8-OHdG) and 8-oxodeoxyguanosine (8-OxodG), and the mechanism of 8-OHdG measurement with ELISA.
