# Supplementary material for: The Effect of β-Carotene, Tocopherols and Ascorbic Acid as Anti-Oxidant Molecules on Human and Animal In Vitro/In Vivo Studies: A Review of Research Design and Analytical Techniques Used
Source: Biomolecules. 2022 Aug 7;12(8):1087. doi: 10.3390/biom12081087 (PMC9406122; doi:10.3390/biom12081087)
Supplement: Supplementary file 1 [file biomolecules-12-01087-s001.zip › biomolecules-1788099-supplementary.pdf]

The Effect of β-Carotene, Tocopherols and Ascorbic acid as Anti-oxidant Molecules on Human and Animal in vitro/in vivo Studies: a Review of Research Design and Analytical Techniques Used

Krystian Miazek, Karolina Beton, Agnieszka Śliwińska and Beata Brożek-Płuska

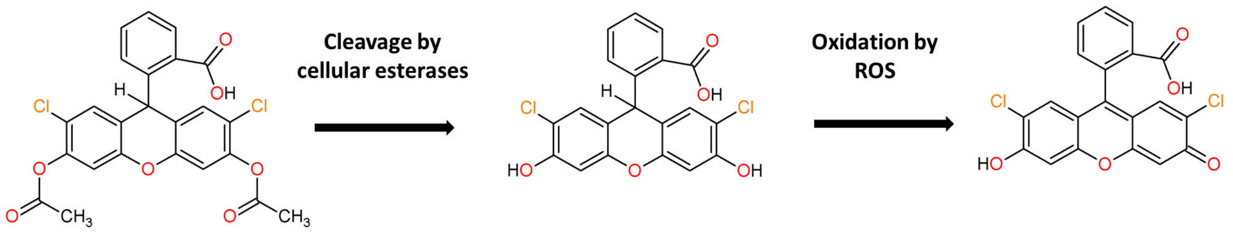

DCFH-DA Non-fluorescent | DCFH Non-fluorescent | DCF Fluorescent

Figure S1. The mechanism of DCFH-DA conversion to DCFH and DCF.

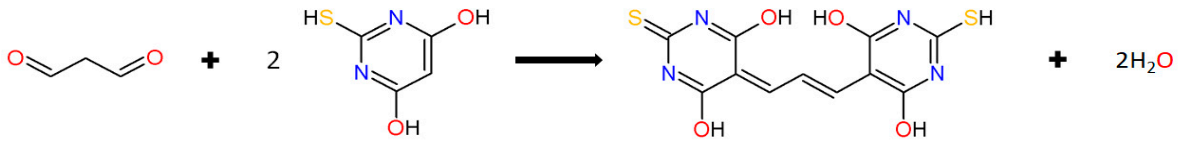

MDA | TBA | MDA-TBA2

Figure S2. The mechanism of reaction between MDA and TBA to form MDA-TBA2.

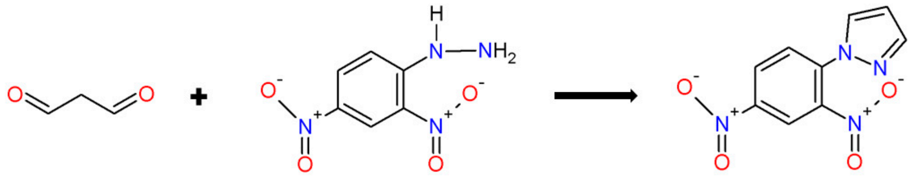

MDA | DNPH | MDA-DNPH

Figure S3. The mechanism of reaction between MDA and DNPH to form MDA-DNPH.

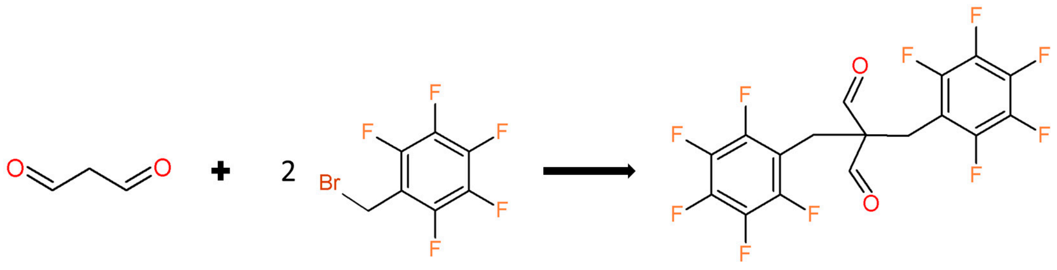

MDA **PFBB** **MDA-PFB2**

Figure S4. The mechanism of reaction between MDA and PFBB to form MDA-PFB2.

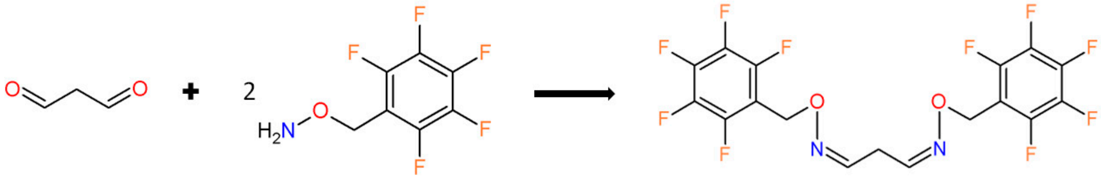

MDA **PFBHA** **MDA-PFB2-oxime**

Figure S5. The mechanism of reaction between MDA and PFBHA to form MDA-PFB2-oxime.

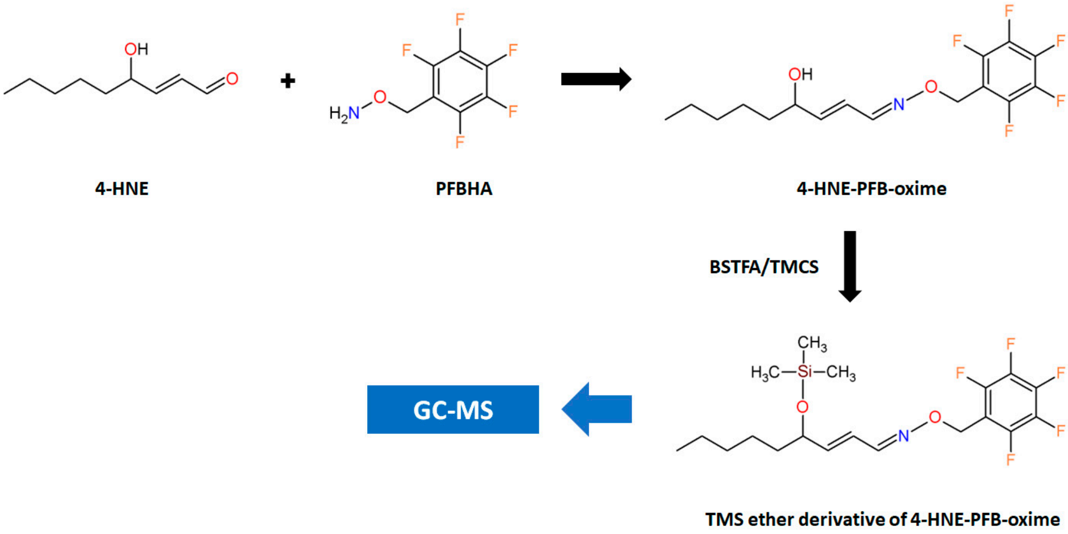

Figure S6. The mechanism of 4-HNE-PFB-oxime-TMS formation and analysis. PFBHA: pentafluorobenzyl hydroxylamine; BSTFA: N,O-bis(trimethylsilyl)trifluoroacetamide; TMCS: trimethylchlorosilane; TMS: trimethylsilyl.

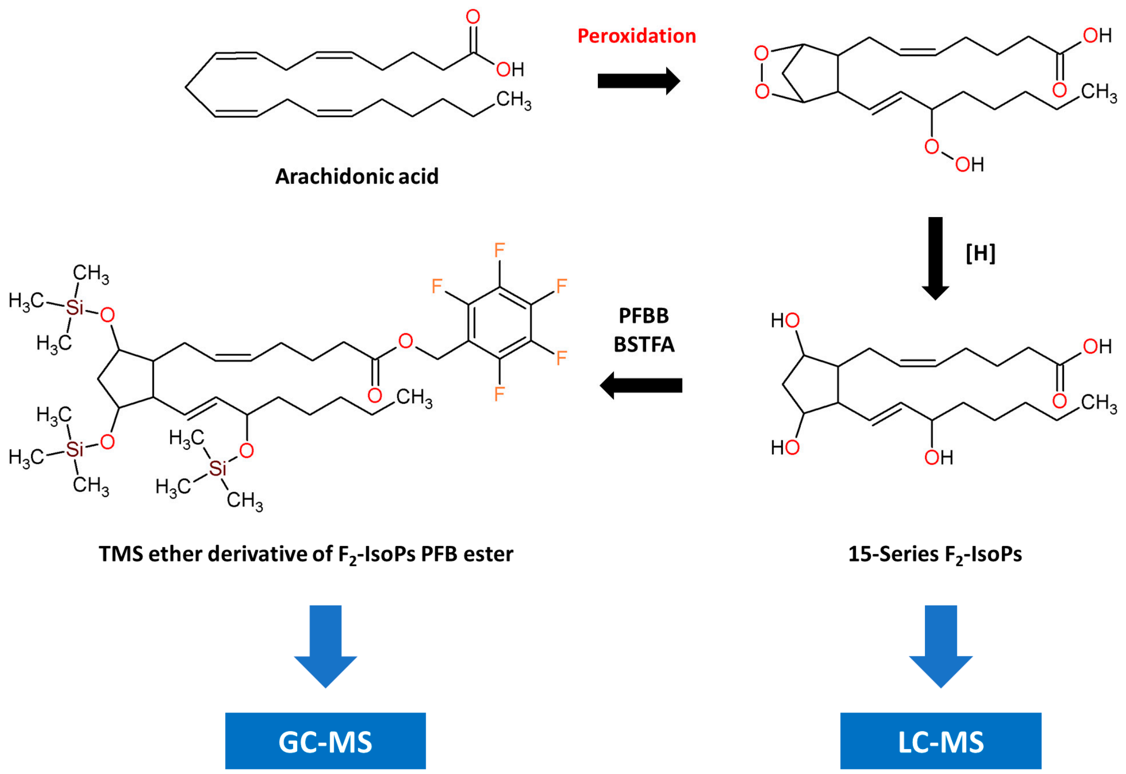

Figure S7. The mechanism of F2-isoprostane (F2-IsoPs) formation and analysis. PFBB: pentafluorobenzyl bromide; BSTFA: N,O-bis(trimethylsilyl)trifluoroacetamide; TMS: trimethylsilyl.

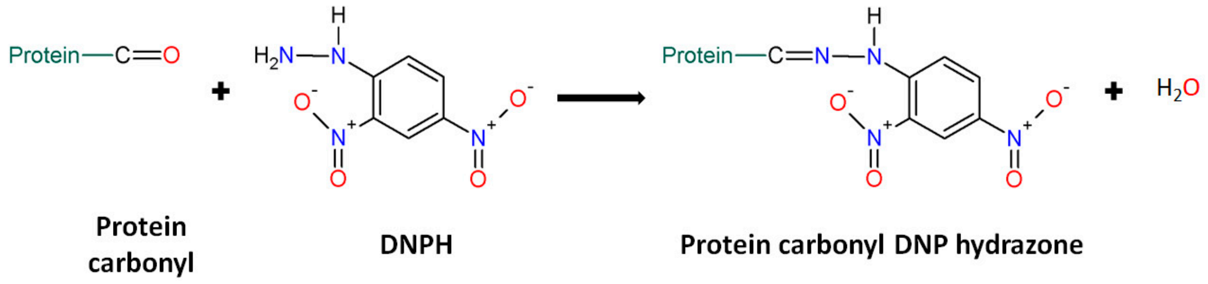

Figure S8. The mechanism of protein carbonyl reaction with dinitrophenylhydrazine (DNPH).

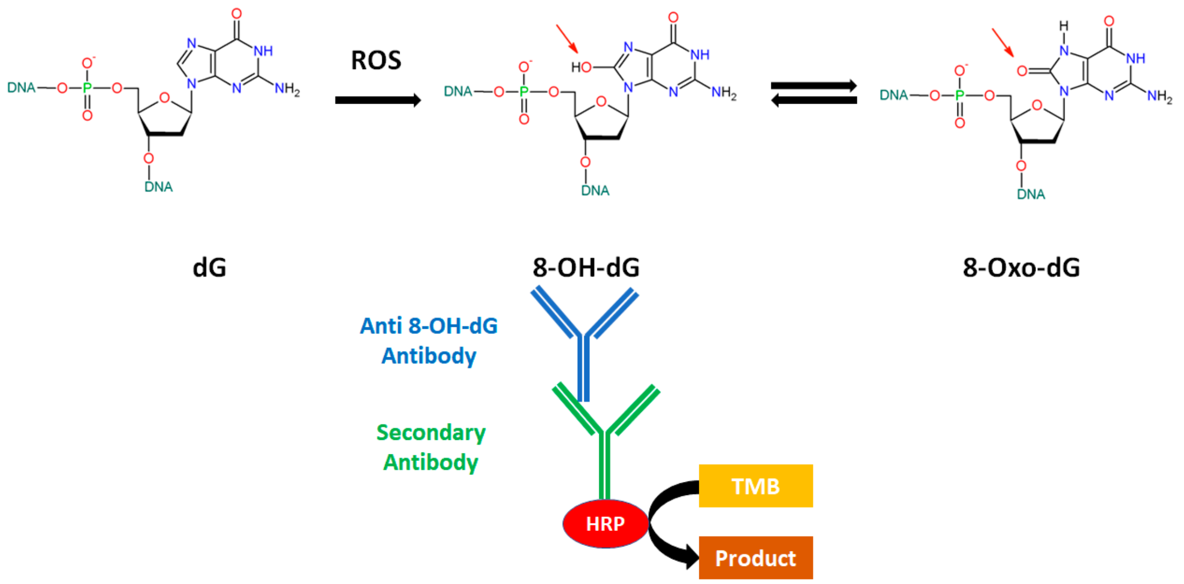

Figure S9. The mechanism of deoxyguanosine (dG) oxidation into 8-hydroxydeoxyguanosine (8-OHdG) and 8-oxodeoxyguanosine (8-OxodG), and the mechanism of 8-OHdG measurement with ELISA.
